# Implementation of singing groups for postnatal depression: experiences of participants and professional stakeholders in the SHAPER-PND randomised controlled trial

**DOI:** 10.3389/frhs.2025.1582517

**Published:** 2025-07-04

**Authors:** Emeline Han, Rachel Davis, Tayana Soukup, Alexandra Bradbury, Julie Williams, Maria Baldellou Lopez, Lorna Greenwood, Rebecca Bind, Carolina Estevao, Tim Osborn, Hannah Dye, Kristi Priestley, Lavinia Rebecchini, Katie Hazelgrove, Manomani Manoharan, Anthony Woods, Nikki Crane, Andy Healey, Paola Dazzan, Nick Sevdalis, Carmine M. Pariante, Daisy Fancourt, Ioannis Bakolis, Alexandra Burton

**Affiliations:** ^1^ Department of Behavioural Science and Health, University College London, London, United Kingdom; ^2^ Centre for Implementation Science, King’s College London, London, United Kingdom; ^3^ Evidera, INC. PPD, Part of Thermofisher Scientific, London, United Kingdom; ^4^ Department of Surgery and Cancer, Faculty of Medicine, Imperial College London, London, United Kingdom; ^5^ Breathe Arts Health Research, London, United Kingdom; ^6^ Department of Psychological Medicine, King’s College London, Institute of Psychiatry, Psychology and Neuroscience, London, United Kingdom; ^7^ South London and Maudsley NHS Foundation Trust, London, United Kingdom; ^8^ Culture Team, King’s College London, London, United Kingdom; ^9^ Centre for Behavioural and Implementation Science Interventions, Yong Loo Lin School of Medicine, National University of Singapore, Singapore, Singapore; ^10^ Department of Biostatistics and Health Informatics, King’s College London, London, United Kingdom

**Keywords:** postnatal depression, group singing, arts in health, creative health interventions, implementation outcomes

## Abstract

**Background:**

There is a rapidly growing evidence base for the effectiveness of creative health interventions in improving mental health, but few studies have explored implementation and scaling of these interventions. The aim of this study was to evaluate the perceived acceptability, appropriateness, and feasibility of a ten-week singing group programme (Breathe Melodies for Mums (M4M)) for mothers experiencing symptoms of postnatal depression (PND) and their babies as well as the programme ingredients that affected these implementation outcomes.

**Methods:**

A mixed methods design was adopted. Quantitative data was collected via the Acceptability of Intervention Measure (AIM), Intervention Appropriateness Measure (IAM), and Feasibility of Intervention Measure (FIM) from 109 intervention participants at 6, 20, and 36 weeks and analysed descriptively. Qualitative semi-structured interviews were conducted with 22 programme participants and 15 professional stakeholders involved in implementing the programme. Qualitative data were analysed using framework analysis.

**Results:**

Quantitative results showed high levels of acceptability, appropriateness, and feasibility among M4M participants, with median scores of 5/5 achieved on the AIM, IAM and FIM at 20 and 36-week follow up. Qualitative results gave insights into the ingredients of M4M that made the programme acceptable, appropriate, and feasible to participants and professional stakeholders. These included “project” ingredients (dose, design, content), “people” ingredients (social composition, activity facilitation), and to a lesser extent, “context” ingredients (setting, project set-up). While participant and stakeholder experiences were largely positive, some challenges and suggestions for improvement were also identified, including broadening recruitment strategies to reach more women.

**Conclusion:**

M4M was highly acceptable, appropriate, and feasible to participants and stakeholders. By identifying the “core” ingredients that facilitated implementation success and strategies to address implementation barriers, these findings have important implications for future implementation and scale-up of M4M and similar creative health programmes.

**Clinical Trial Registration:**

identifier (NCT04834622).

## 1 Background

Postnatal depression (PND) affects around 20% of mothers following childbirth and can manifest as low mood, anxiety, feelings of worthlessness and guilt, as well as impaired concentration and memory (1). These symptoms can have serious effects on family health, with untreated PND being linked to maternal suicide (2, 3) and poorer child developmental outcomes (4, 5). Common risk factors for PND include low perceived social support, exposure to traumatic events during pregnancy and childbirth, and high stress associated with childcare (1, 6, 7). In recent years, these factors have been exacerbated by the COVID-19 pandemic, due to social distancing restrictions, increased risk of domestic violence (8), as well as reduced postnatal support and childcare options (6). A population-based study in England found that almost half (47.5%) of new mothers met the threshold for PND during the first COVID-19 lockdown (9). This prevalence rate was more than double the European average (23%) before the pandemic (10).

The high prevalence and potentially severe impact of PND underscore the need for timely and effective treatment pathways. While research suggests that anti-depressants can be effective at reducing PND symptoms, uptake and adherence are low due to concerns about adverse side effects on mothers and their babies through breastfeeding (11, 12). Mothers across cultures have shown a preference for non-pharmacological treatments (13), and systematic reviews have found that psychological therapies are viable for treating PND (14–16). However, access to such treatments is often hindered by stigma, overstretched and fragmented services, as well as language, cultural, and socioeconomic barriers (17, 18).

Creative health interventions are gaining traction as a way of supporting maternal mental health (19, 20), with evidence to show that group singing and music making can alleviate depression and anxiety in new mothers (21, 22). One such intervention is Melodies for Mums (M4M), a 10-week group singing programme for mothers with PND that has been delivered by Breathe Arts Health Research since 2017. M4M was developed based on an earlier randomised controlled trial (RCT), which found that women with moderate to severe PND symptoms who participated in group singing recovered significantly faster than those in creative play and usual care groups (23). Subsequently, a hybrid type 2 RCT designed to assess both clinical effectiveness and implementation outcomes was conducted to evaluate M4M at scale (SHAPER-PND) (24, 25). This trial was paused during the COVID-19 pandemic and an online study was rapidly set up, with evidence of antidepressant efficacy (26). The main trial resumed after the pandemic and completed in June 2024. Main trial results suggest that M4M participants had more lasting improvements in PND symptoms that continued up to six months after the intervention, while the active control group plateaued after 10 weeks (27). The clinical outcomes of M4M will be detailed in a separate paper, with this paper reporting on its implementation outcomes.

Implementation outcomes serve as preconditions for attaining desired clinical outcomes and indicators of implementation success, with perceived acceptability, appropriateness, and feasibility by intervention stakeholders often used as “leading indicators” (28, 29). While acceptability, appropriateness, and feasibility are conceptually distinct, it is recognised that they are likely to be “empirically inter-related in complex ways” (28, 29). This complexity is further compounded by the multiple interacting components, also termed “active ingredients”, in complex interventions such as M4M (30). Active ingredients refer to the specific components of an intervention (*what* it consists of) that activate mechanisms of change (*how* the intervention works) (30). While there is increasing insight into the mechanisms and outcomes of creative health interventions, inadequate and inconsistent reporting of their active ingredients limit validity and replicability (31). To address this limitation, a comprehensive theoretical framework, INgredients iN ArTs in hEalth (INNATE), has been specifically developed to support the identification and categorisation of active ingredients of creative health interventions (32). Previous research has suggested that group singing may relieve PND symptoms by facilitating a sense of achievement, mother-infant bonding, and social connection with other mothers (21, 22). However, it remains unclear which active ingredients drive these changes. It is important to understand which ingredients of M4M are considered essential for implementation success to inform continued delivery and future scale-up of the programme. Thus, the aims of this study were to (i) evaluate the perceived acceptability, appropriateness, and feasibility of M4M among programme participants and professional stakeholders, and (ii) identify the key active ingredients affecting these implementation outcomes using the INNATE framework.

## 2 Methods

### 2.1 Study design

SHAPER-PND is a two-arm hybrid type 2 trial (33) assessing both the clinical and implementation outcomes of M4M. This study design was chosen as M4M had demonstrated clinical effectiveness in a smaller trial (23), but important questions remained around its clinical impact and implementation processes on a larger scale. Quantitative data on the implementation effectiveness of M4M was collected using the Acceptability of Intervention Measure (AIM), Intervention Appropriateness Measure (IAM), and Feasibility of Intervention Measure (FIM) (25). Qualitative interviews were conducted to explore factors affecting the perceived acceptability, appropriateness, and feasibility of M4M (Table 1).

**Table 1:** Definitions of implementation outcomes as applied to M4M.

Implementation outcomes	Definitions as applied to M4M (28)
Acceptability	The perception among participants and professional stakeholders that M4M is agreeable, palatable and satisfactory.
Appropriateness	The perception among participants and professional stakeholders that M4M is fit, relevant and suitable for the management of PND.
Feasibility	The perception that M4M is practically doable and can be successfully used (by participants) or delivered (by professional stakeholders).

### 2.2 Intervention

M4M is a 10-week group singing programme for new mothers experiencing symptoms of PND. Sessions are led by a specialist Breathe-trained music lead supported by Breathe staff members who provide regular briefing, debriefing and training opportunities focused on their practice but within the context of working with participants with PND. Supporting Breathe staff members are experienced in working with vulnerable people and trained in safeguarding. M4M is usually delivered in-person to groups of 8–15 mothers and their babies. For SHAPER-PND, M4M sessions were delivered in-person to groups of 3–13 mothers and their babies; initial challenges with recruitment led to smaller groups being run at the beginning of the trial. Sessions are free to attend and for SHAPER-PND, took place in Children & Family Centres in Southwark, Lambeth and Lewisham. Sessions last 1 h each and start with physical and vocal warm-ups, welcome songs and introducing participants to one another, followed by a range of singing activities in different styles and languages. While sessions across groups and locations follow this general structure, singing leads may adapt their delivery by providing different ways for mothers to engage depending on their level of confidence (e.g., introducing harmonies, rounds, and simple instruments). There is also time to socialise and provision of snacks and drinks after the sessions. Participants have access to a library of song recordings to listen to at home, lyrics and signposting links to additional mental health support provided by charitable organisations. Additionally, participants are offered access to a WhatsApp group moderated by a Breathe staff member. Supplementary File 1 contains the TIDieR checklist and Supplementary File 2 contains the INNATE checklist completed by LG, DF, and AB, which details the full programme ingredients of M4M.

### 2.3 Recruitment

Participants were recruited to the SHAPER-PND trial between September 2021 and December 2023 through: (1) signposting or referral via health and social care professionals; (2) flyers and posters at baby weighing clinics and community clinics for postnatal mothers; (3) social media and online platforms aimed at new mothers; (4) GP practice mail-outs coordinated by the National Institute for Health Research (NIHR) Clinical Research Network (CRN), and (5) word-of-mouth. All participants had to register themselves for the study via the Breathe website. Mothers were eligible for the trial if they were: able to understand English and give informed consent, over 18 years old, had a baby 0–9 months old, and reported symptoms of PND (scoring ≥10 on the Edinburgh Postnatal Depression Scale (EPDS) in an online screening form when registering with Breathe). The main trial paper contains further details on how eligibility was assessed (27). 199 mothers were recruited and randomly allocated to receive either M4M (intervention, *n* = 133) or signposted to non-music groups in the community (control, *n* = 66).

Quantitative implementation surveys were sent to all participants in the M4M and control groups. This paper only reports the intervention findings; comparisons between M4M and the control group and cost effectiveness analyses are reported in a separate paper (27). For the qualitative interviews, all participants who took part in the first three of seven singing groups were invited to take part via email. Recruitment ceased when data saturation was reached, i.e., when the researchers RD, TS, and MBL discussed and agreed that no new themes were being discussed in further interviews. Additionally, professional stakeholders were recruited through Breathe's network of artists, staff and others involved in implementing M4M.

### 2.4 Data collection

The AIM, IAM, and FIM have shown high validity and reliability in prior psychometric assessments in other mental health intervention studies (29, 34, 35). Each measure has four items and scale values ranging from 1 to 5, with higher scores indicating higher perceived acceptability, appropriateness, and feasibility. Examples of items include “I like [the intervention]” (AIM), “[the intervention] seems suitable” (IAM), and “[the intervention] seems doable” (FIM). All three measures were administered to M4M participants at three timepoints: 6 weeks, 20 weeks, and 36 weeks via online questionnaires sent to participants by email.

Qualitative data was collected using semi-structured interviews between March and September 2022 within a few weeks after each singing programme had ended. Interviews were conducted by RD (a female research scientist (PhD, MSc, BSc) with a background in health psychology and implementation science, experience of being a mother and interviewing patient and professional stakeholder groups) or MBL (a female medical student (MSc, BSc) with experience in neuropsychology research) via Zoom and lasted between 12 and 56 min (average 25 min). Babies were often present with their mothers during the interviews. All interviews followed a topic guide developed by TS and RD and informed by topic guides used in previous hybrid trials (36, 37). Participants were all asked the same questions, while questions were adapted for professional stakeholders depending on their role (see Supplementary File 3 for topic guides and Supplementary File 4 for the COREQ checklist). All interviews were audio- or video-recorded, de-identified and transcribed verbatim by an external transcription company called Page Six.

### 2.5 Data analysis

Quantitative data was analysed descriptively. Scores on the AIM, IAM and FIM were summed and averaged for each measure, with medians and interquartile ranges, minimum and maximum scores reported respectively.

Qualitative data was analysed using framework analysis following the five steps of familiarisation, identifying a framework, indexing, charting, mapping and interpretation (38). After familiarisation, the interview topic guide was first used to create an initial coding framework using NVivo12 software, with new codes and sub-codes added as transcripts were indexed. All transcripts were coded by either TS, RD, or MBL and then reviewed for consistency. Coding discrepancies were resolved through discussion until consensus was established. Next, the charting process involved EH, ABr and AB summarising the indexed data and organising it according to the “project”, “people”, and “context” categories of the INNATE framework (Table 2). Subsequent mapping and interpretation focused on identifying the specific ingredients that contributed to the acceptability, appropriateness, and feasibility of M4M as defined in Table 1.

**Table 2:** Definitions of INNATE categories as applied to M4M.

INNATE categories	Definitions as applied to M4M (32)
Project	Active ingredients in the “project” category relate to the *attributes* (e.g., format, dose, design, content) of M4M, as well as the kinds of stimuli involved in prompting *engagement* with M4M.
People	Active ingredients in the “people” category denote *social composition*, relating to how people interact through engagement with M4M and who is involved in this interaction, as well as the *activity facilitation*, concerning the people who lead, guide, or facilitate the participant-facing aspects of M4M.
Context	Active ingredients in the “context” category relate to the activity *setting,* comprising the place(s), things, surroundings and feelings that make up the situation, as well as *project set-up*, such as the structure, processes and/or systems which surround the delivery of M4M.

## 3 Results

### 3.1 Participant characteristics

Of the 133 intervention participants, 109 participated in the quantitative implementation surveys. Two participants withdrew from the study (without providing reasons), 1 did not attend any M4M sessions and 21 did not complete the surveys for unknown reasons. Of the 42 women approached for qualitative interviews, 13 did not respond, 2 refused because they did not want to be recorded, 1 was out of the country at the time of recruitment, 1 did not attend any M4M sessions, and 3 declined or did not turn up for the interview. Of the 22 women interviewed, 11 had been recruited to the main trial through signposting or referral via health and social care professionals, 7 via word-of-mouth, and 4 via social media. Table 3 shows the characteristics of the programme participants who took part in the M4M intervention, quantitative surveys, and qualitative interviews respectively.

**Table 3:** M4m participant socio-demographic characteristics.

Characteristics	Intervention participants (*n* = 133)	Survey participants (*n* = 109)	Interview participants (*n* = 22)
Age (Mean, range)	35.6 (22–47)	35.5 (22–47)	36.6 (30–44)
Ethnicity			
White	93 (69.9%)	78 (71.6%)	17 (77.3%)
Black	14 (10.5%)	9 (8.3%)	2 (9.1%)
Asian	12 (9.0%)	9 (8.3%)	0 (0.0%)
Mixed ethnicity	12 (9.0%)	11 (10.1%)	3 (13.6%)
Other ethnicity	2 (1.5%)	2 (1.8%)	0 (0.0%)
English as first language	103 (77.4%)	82 (75.2%)	20 (90.9%)
Marital/living status			
Married/cohabiting	115 (86.5%)	94 (86.2%)	19 (86.4%)
Single (no partner)	18 (13.5%)	15 (13.8%)	3 (13.6%)
Educational qualifications			
Degree/diploma	113 (85.0%)	95 (87.2%)	21 (95.5%)
GCSEs/A levels	19 (14.3%)	14 (12.8%)	1 (4.5%)
Employment status			
Employed/maternity leave	112 (84.2%)	100 (91.7%)	19 (86.4%)
Unemployed/student	21 (15.8%)	9 (8.3%)	3 (13.6%)
Income bracket			
>£30k (above national average)	110 (82.7%)	93 (85.3%)	20 (90.9%)
<£30k (below national average)	17 (12.8%)	12 (11.0%)	1 (4.5%)
M4M Attendance			
>50%	117 (88.0%)	106 (97.2%)	20 (90.9%)
<50%	16 (12.0%)	3 (2.8%)	1 (4.5%)

Of the 23 professional stakeholders invited to interview, 5 did not respond and 3 declined to participate as they were not directly involved in programme implementation. Table 4 shows the roles of the 15 stakeholders who were interviewed.

**Table 4:** Professional stakeholder characteristics.

Role in M4M	Role description	*n* (%)
Referrer	Referring or signposting participants to M4M via the NHS or Children & Family centres	6 (40%)
Breathe staff	Providing oversight and coordination of M4M delivery (project manager), or administrative and pastoral support to M4M sessions (support officer)	5 (33%)
Music lead	Delivering M4M sessions	4 (27%)

### 3.2 Quantitative results

M4M was rated as highly acceptable (4.75/5), appropriate (4.25/5), and feasible (4.75/5) by participants at week 6, with median scores on the AIM, IAM, and FIM increasing to 5/5 at 20- and 36-week follow-up (Table 5).

**Table 5:** Medians, interquartile ranges and minimum/maximum scores for implementation outcomes at 6, 20, and 36 weeks among M4M participants (*n* = 109).

Measure	6 weeks		20 weeks		36 weeks	
	*N*	Median (IQR, min–max)	*n*	Median (IQR, min–max)	*n*	Median (IQR, min–max)
AIM	109	4.75 (4.25–5, 1–5)	87	5 (4.75–5, 1–5)	88	5 (4.75–5, 1–5)
IAM	109	4.25 (4–5, 1–5)	87	5 (4.25–5, 1–5)	88	5 (4–5, 1–5)
FIM	109	4.75 (4–5, 1–5)	87	5 (4.25–5, 1–5)	88	5 (4–5, 1–5)

**Figure 1:**
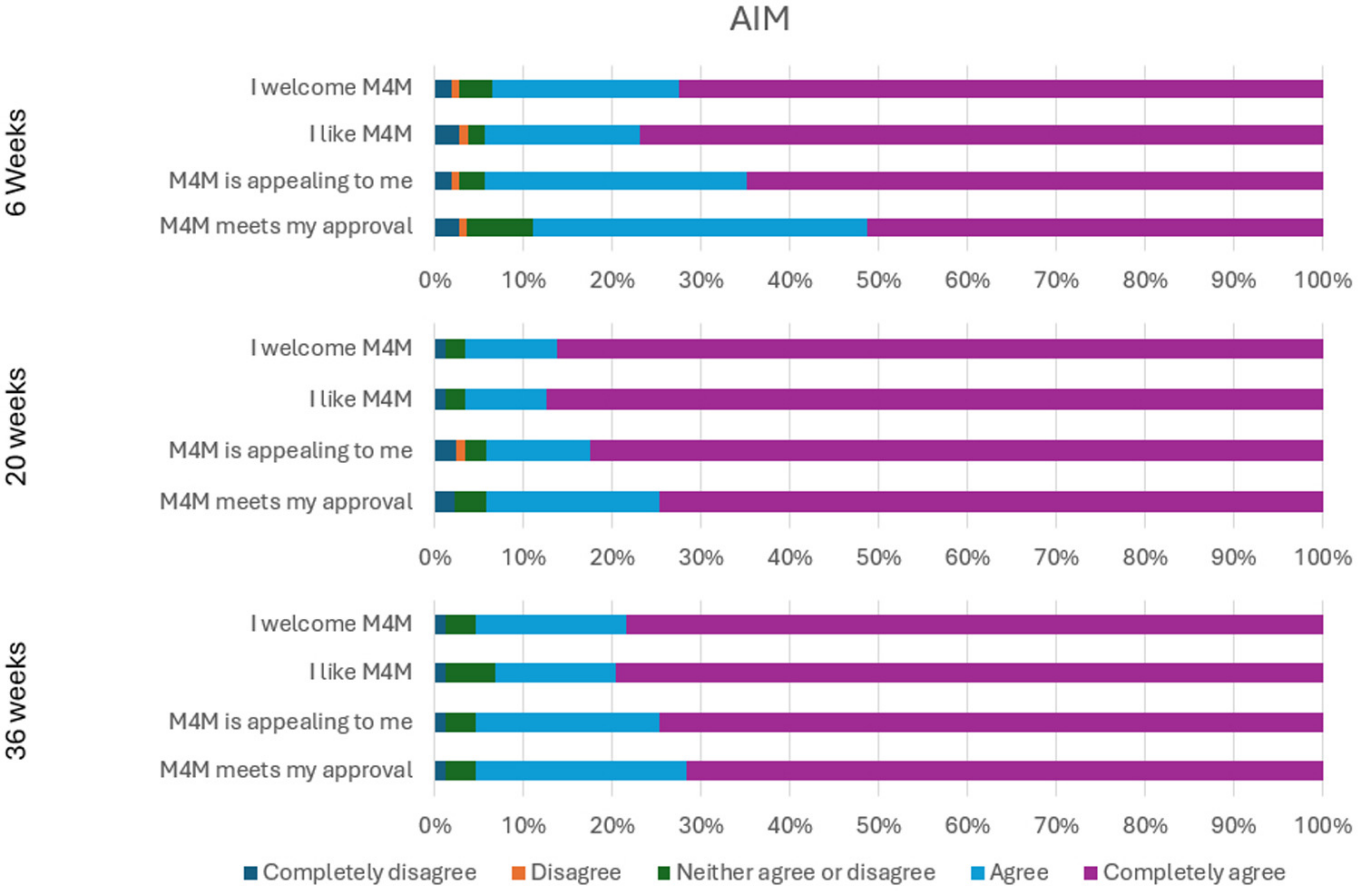
AIM responses at weeks 6, 20, 36.

**Figure 2:**
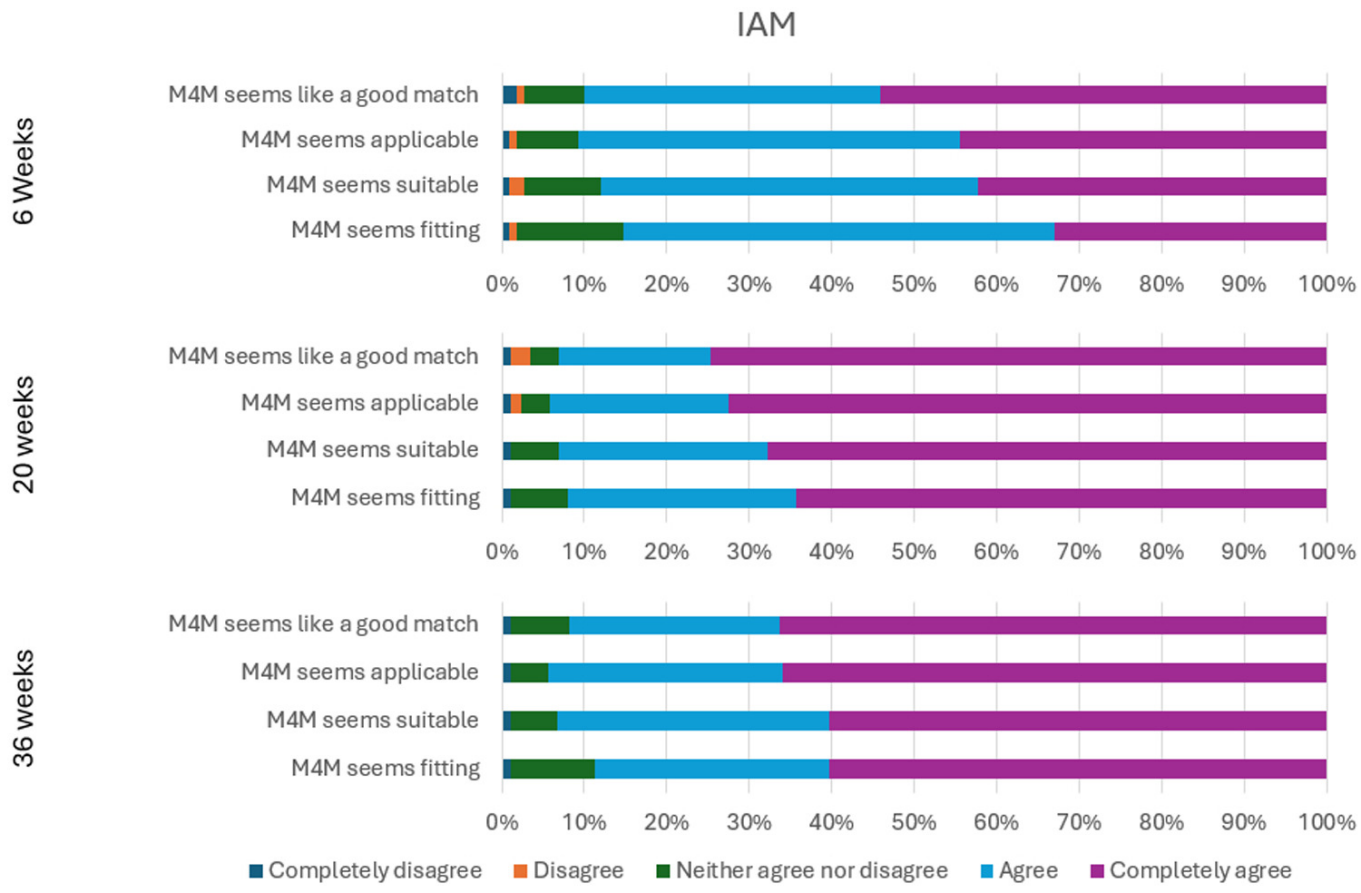
IAM responses at weeks 6, 20, 36.

**Figure 3:**
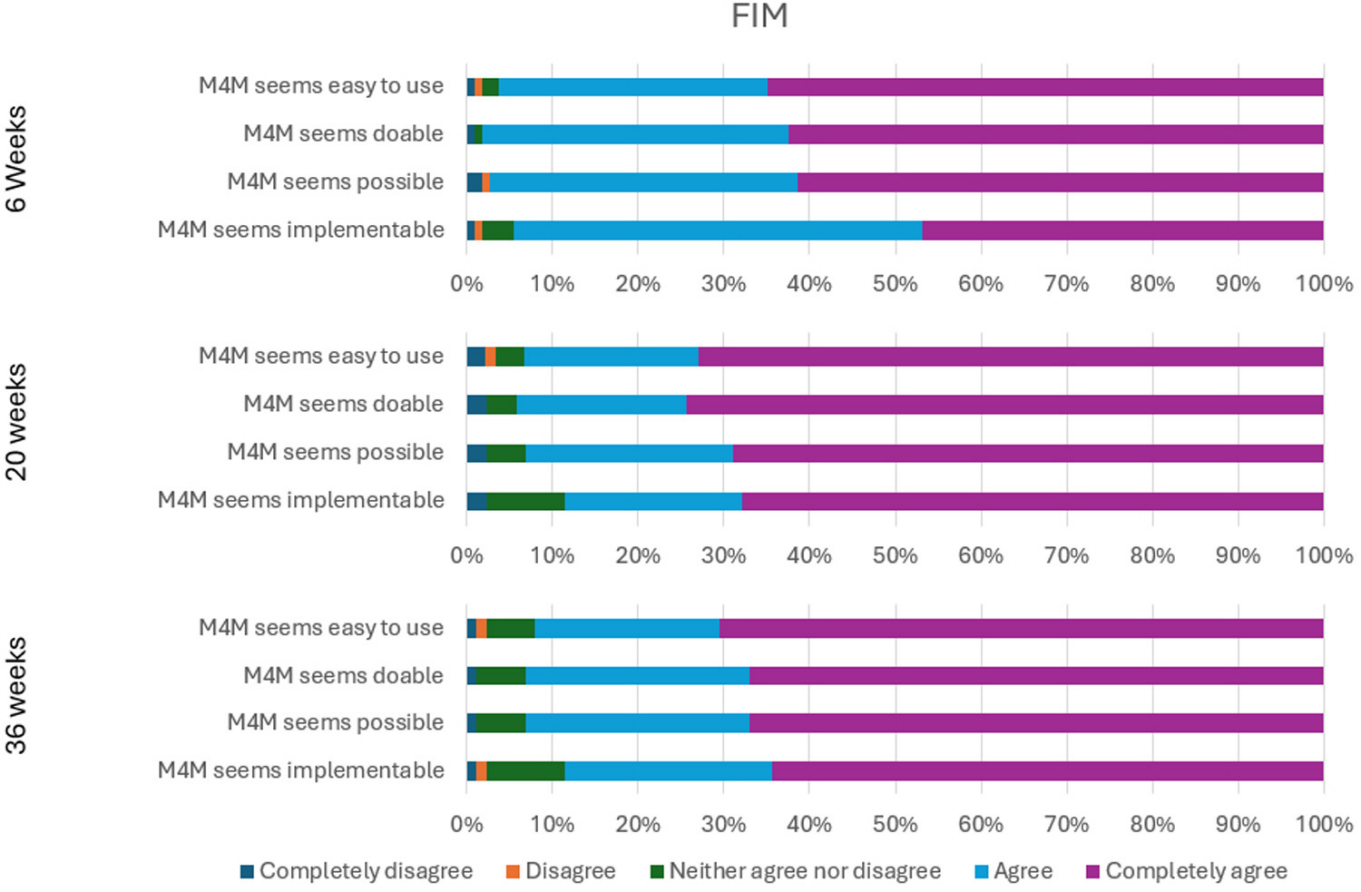
FIM responses at weeks 6, 20, 36.

All items on the AIM, IAM, and FIM received a minimum of 85.3% agreement and a maximum of 3.7% disagreement at all time points. These consistent and sustained positive perceptions over time suggest strong endorsement of the acceptability, appropriateness, and feasibility of the intervention, supporting the case for wider implementation. Figures 1–3 show the percentage of responses to each item on the three measures at Weeks 6, 20, and 36 respectively.

### 3.3 Qualitative results

Sixteen core programme ingredients were identified that affected the perceived acceptability, appropriateness and feasibility of M4M. Six ingredients related to the “project” aspects of M4M, seven related to the “people” aspects of M4M, and three related to the “context” in which M4M was delivered. Table 6 indicates where M4M participants, stakeholders or both identified the respective ingredients as acceptable, appropriate, or feasible. Blank cells refer to an ingredient not being discussed as acceptable, appropriate, or feasible by either the participants or stakeholders. Each ingredient is then described in detail and supported by anonymised quotes from M4M participants (M) and stakeholders (S). A supplementary table of additional quotes is available in Supplementary File 5.

**Table 6:** Programme ingredients identified as acceptable, appropriate and feasible by participants and professional stakeholders.

Category	Sub-category	Ingredient	Participant			Professional stakeholder		
			Acceptable	Appropriate	Feasible	Acceptable	Appropriate	Feasible
Project	Dose	Frequency of sessions		✓				
		Length and number of sessions		✓	✓		✓	✓
	Design	Flexible and adaptable structure	✓			✓		
		Difficulty level of musical activities	✓	✓		✓	✓	
	Artistic content	Diverse range of songs	✓	✓		✓		
		Singing together as a group	✓	✓		✓	✓	
People	Social composition	Small group size	✓	✓			✓	
		Shared mental health experiences among participants	✓	✓			✓	
		Shared activity between mother and baby	✓	✓				
		Structured social time during sessions		✓			✓	
		Unstructured social time outside of sessions		✓			✓	
	Activity facilitation	Skills and values of music lead	✓					✓
		Support from additional staff	✓					✓
Context	Setting	Location and transport			✓			✓
		Time and day of sessions			✓			✓
	Project set-up	Recruitment and advertising	✓			✓		

#### 3.3.1 Project

Six ingredients related to the “project” aspects of M4M—these are sub-categorised into the dose, design, and artistic content of the programme.

##### 3.3.1.1 Dose

Two ingredients related to the dose of M4M (i.e., the amount of activity received by participants), including the frequency, length, and number of sessions.

###### 3.3.1.1.1 Frequency of sessions

M4M participants felt that the frequency of sessions was **appropriate** as it provided a new sense of routine. The weekly sessions gave mothers a “*bright spot to look forward to*”, a “*goalpost*” to work towards, and a “*reason to get out of the house*” when they lacked motivation. Through attending M4M regularly, some women said that they built confidence to travel with their babies, attend other mother and baby groups, or meet up with friends:

“After having [X] I was having really bad panic attacks and I couldn’t really go anywhere…that was the first time I’ve got into London again and doing that every week I would then make plans to meet a friend in town after the class and from there got back into travelling.” (M19)

###### 3.3.1.1.2 Length and number of sessions

Both M4M participants and stakeholders felt that one hour was an **appropriate** amount of session time to facilitate a sense of achievement as well as **feasible** for babies' attention spans:

“I think they were perfect because I always felt we got so much out of the session and then it was just the right amount before babies start getting grisly, it held their attention for the right amount of time. And, then we would both go away feeling ‘oh we really achieved something today’ and yes I thought it was spot on!” (M9)

Similarly, M4M participants and stakeholders felt that the 10-week duration was **appropriate** to facilitate group bonding and a change in PND symptoms. While some participants and stakeholders wished the programme could be longer or ongoing, they acknowledged that it may not have been **feasible** for them to commit to more sessions:

“It always feels like it could be longer. You get to the end, and it feels…quite sad…But I think it feels about right and I think probably for leaders it’s a manageable chunk…it’s not manageable for me to commit to 20 weeks of something.” (S2)

These quotes suggest that the 10-week length effectively balanced practical feasibility with therapeutic depth.

##### 3.3.1.2 Design

Two ingredients related to the design (i.e., structural plan) of the activity. These included a flexible and adaptable structure and the difficulty level of the musical activities.

###### 3.3.1.2.1 Flexible and adaptable structure

While sessions followed a broad structure, handbook and library of songs, M4M participants reported that the flexibility in delivery made M4M **acceptable** and facilitated their enjoyment. Mothers appreciated that there was no pressure to cover all the material planned for each session, and they could arrive early or step out of sessions to feed and change their babies:

“You don’t feel like we’ve already started ten minutes late and we need to start doing the first song, it wasn’t like that, it was just let’s check in with everybody and see where we’re at and maybe we do two less songs today, but it doesn’t matter.” (M18)

Project managers shared that they did not interfere with the way sessions were delivered, allowing music leads to adapt their delivery according to the needs of the group. While stakeholders felt that this flexible approach was **acceptable** on a smaller scale as project managers were able to give direct feedback to music leads, they were concerned about how to maintain a level of consistency and quality when rolling out M4M more widely:

“We’ve come up with a structure template for a Melodies for Mums session but it’s got creative wiggle room in it for the music lead. Ideally you’d want to keep that but…how would you roll that out if you’re training up 20 music leads around the country, how do you keep the quality of it?” (S6)

###### 3.3.1.2.2 Difficulty level of musical activities

M4M participants and stakeholders felt that the musical activities in M4M were pitched at an **acceptable** difficulty level and accessible to people with varying musical abilities. Music leads spoke of “*how to creatively challenge these women whilst being careful and gentle with that*”, including easing them in with simpler melodies and then gradually building up to more complex activities like singing in harmonies and changing the lyrics to songs. While most mothers enjoyed the challenge of learning something new, a few struggled to remember lyrics:

“I found it was a very nice pace week by week, but definitely towards the end of the sessions, I think, nothing wrong with the instructor at all, they were amazing, but I just struggled sometimes remembering the words.” (M17)

M4M participants and stakeholders also deemed the musical activities **appropriate** for managing PND as they were “*difficult enough to do that it takes you out of yourself*”, but easy enough to gain a sense of accomplishment:

“I teach very short rounds that are mainly rounds or easy songs that are very easy to teach and learn quickly, so they get the sense of achievement; they are not sitting there having to work [it] out.” (S13)

##### 3.3.1.3 Artistic content

The remaining two “project” ingredients related to the artistic content of M4M and included the diverse range of songs and singing together as a group.

###### 3.3.1.3.1 Diverse range of songs

The diverse range of songs on offer was **acceptable** to M4M participants and stakeholders. Mothers liked that the songs varied in tempo and mood, from “*calming*” to “*upbeat*”, which made the sessions “*both energising and relaxing*”. They also liked that the programme included songs from different countries and languages, giving them the opportunity to “*learn about what motherhood means to different cultures*”. Likewise, stakeholders highlighted the multicultural repertoire as a unique strength of M4M:

“The repertoire of international songs has always been quite strong, and the first musician that brought them in, but we’re always bringing songs, we always invite women who are from other nationalities and cultures, and from their own experiences as well, to bring things in that they’ve liked to sing and they want to share.” (S1)

M4M participants and stakeholders also felt that song choices were **appropriate** for lifting their mood as lyrics centered around positive themes such as “*sunshine, spring or the sun*”. Some mothers shared that the new lullabies they learnt were helpful for calming their babies, which in turn helped to decrease their anxiety and stress:

“I have taken the time to put the songs on a playlist on iTunes which me and my partner have access to, and when she is having a moment, we play the songs we sang and two of the songs send her into a state of relaxation, so yes it has been amazing.” (M9)

These quotes illustrate how the carefully selected songs served as a medium for cultural expression and emotional regulation.

###### 3.3.1.3.2 Singing together as a group

M4M participants and stakeholders affirmed that communal singing was an **appropriate** activity for mothers with PND given its “*uplifting*” and “*cathartic*” nature. Singing in rounds helped one woman to make eye contact with other people instead of socially withdrawing on a bad day, and helped another woman to focus on something other than her anxiety:

“I think the…communal singing aspect made me feel relaxed, it made me feel calm…there’s something about singing, especially when you're doing it as a group in a round you really have to focus on what you're doing. So, it put my head in a different space and helped me switch off from other negative thoughts or anxieties.” (M1)

Most mothers also found singing in a group **acceptable** as they enjoyed working together to “*make something beautiful*” that they could not create by themselves. Both M4M participants and stakeholders found it rewarding to witness the group progress musically:

“I’m getting to the stage now where with some songs I’ll sing a lot quieter and sometimes even stop singing, and you’ve got this lovely collective of voices…I feel I can step away and be like, ‘You guys sound absolutely awesome’.” (S10)

Only one M4M participant found the group singing “*not that musically satisfying*” but acknowledged that this sentiment could be unique to her given her professional singing background.

#### 3.3.2 People

Seven ingredients pertained to the “people” aspects of M4M—these are sub-categorised into social composition and activity facilitation.

##### 3.3.2.1 Social composition

Five ingredients pertained to the social composition of M4M groups (i.e., who was involved in the activity and how they interacted). These included small group size, shared mental health experiences among participants, shared activity between mother and baby, structured social time within sessions, and unstructured social time outside of the sessions.

###### 3.3.2.1.1 Small group size

M4M participants and stakeholders said that the small group size of 3–13 women was **appropriate** to promote social connection as it felt less intimidating, making it easier for mothers to engage with each other and their babies:

“Small group sizes are really important for the bonding of women and bonding with their baby so you’re not in a really loud, overwhelming room.” (S8)

However, a few stakeholders and M4M participants in groups of 6 women or fewer felt that the group size was less **acceptable** for music-making as individuals felt “*more exposed than anticipated*”:

“It was nice on a social element having an intimate group, but you just don’t get quite so much out of the singing.” (M8)

These M4M participants and stakeholders felt that a slightly larger group of 10–12 mothers would have created a more enjoyable singing experience but stressed that the group should not exceed 15 people to avoid losing its “*personal touch*”.

###### 3.3.2.1.2 Shared mental health experiences among participants

M4M participants enjoyed meeting other mothers and valued the social connections they made, which were particularly strong because they were based on shared experiences of motherhood and mental health difficulties. For some, knowing that others were going through similar struggles made the programme **acceptable** as it meant that they could “*be completely open*” rather than pretend that they were “*feeling really cheerful*”:

“It was actually quite nice to know that perhaps these people would particularly understand if maybe I was a bit quiet one week.” (M6)

M4M participants and stakeholders also said that the shared experience made M4M **appropriate** as it created a safe community for mothers with PND to support each other and feel less alone:

“I've seen first-hand for one of my ladies it’s been incredibly helpful, and that’s somebody that’s quite isolated and she just found it a really safe, supportive group.” (S7)

Nonetheless, there were a few M4M participants who felt that their group “*didn*’*t talk to each other that much*”, and stakeholders also mentioned one or two women “*who had huge social anxiety*” or who reported feeling like the “*odd one out*”.

###### 3.3.2.1.3 Shared activity between mother and baby

M4M participants found the programme **acceptable** because it “*equally included*” them and their babies, rather than being a “*babies’ group*” or “*mums’ group*”. Mothers expressed the joy of introducing their babies to music and seeing them participate in sessions:

“I enjoyed it most when he was awake and really, really enjoying it and getting involved which he was for some of the sessions.” (M8)

M4M as a shared activity also made it **appropriate** because babies would recognise songs from M4M and respond positively when mothers sang to them at home. As a result, mothers felt better able to engage with their babies:

“When I talk to my daughter, back in the day, sometimes she wouldn’t look at me, or make eye contact, which made me nervous, and as soon as I went to [M4M] and then I sang at home with her, she would make eye contact, and that was amazing.” (M17)

###### 3.3.2.1.4 Structured social time during sessions

M4M participants and stakeholders felt that it was **appropriate** to have structured social time during sessions as it gave mothers the opportunity to share their feelings and bond with each other:

“The leader…always made time at the beginning for us to go round and just say where we’re at and what kind of week we’ve had, and I think that was really nice.” (M18)

Some M4M participants wished that sessions could incorporate more social time, especially because they had limited opportunities for social interaction since the pandemic. Stakeholders described a tension between wanting to allow more time for socialising but also making clear that M4M was a singing group and “*not a therapy group*”:

“You don’t want to stifle it, but at the same time, the focus of the session is on singing.” (S1)

###### 3.3.2.1.5 Unstructured social time outside of sessions

In addition to integrated social time, M4M participants and stakeholders conveyed that M4M delivery staff facilitated unstructured social time outside of sessions that was **appropriate** to support mothers with PND. Stakeholders actively encouraged participants to meet up and created a WhatsApp group that became part of their “*social support network*”:

“There was a WhatsApp group that was set up with the other mums so we could keep in contact after the sessions and meet up and go for walk…that was really positive.” (M14)

For some mothers, these external interactions led to friendships that continued even after the programme ended, while others found it challenging to sustain friendships due to geographical and time constraints:

“We have best intentions to keep in touch and we did meet up once but they’re a bit far for me…when you have a baby, your circle just really shrinks.” (M22)

##### 3.3.2.2 Activity facilitation

The final two “people” ingredients pertained to activity facilitation (i.e., the people involved in facilitating M4M and their facilitation style), including the skills and values of the music lead and support from additional staff.

###### 3.3.2.2.1 Skills and values of music lead

M4M participants said the programme was **acceptable** because music leads were inclusive, empathetic, and encouraging, especially towards mothers with less experience or confidence in singing:

“The teacher was so lovely about everyone singing and that made me feel a bit more confident as well…I just didn’t feel weird or self-conscious that I didn’t have any experience at all.” (M15)

Similarly, stakeholders said it was **feasible** to implement M4M because music leads not only had musical expertise, but also the “*soft skills*” to sensitively manage mothers' emotions:

“It is about the soft skills of the practitioner, to be honest…just because you are [a] musician doesn’t mean that you have the soft skills to look after vulnerable people, it doesn’t mean that you would necessarily get the boundaries of relationships appropriately in place.” (S9)

Given the dual role of facilitators as artists and informal supporters, safeguarding procedures and training for music leads to equip them with an “*understanding of arts in health and the needs of people with postnatal depression*” were seen as vital to implementing and scaling up M4M successfully.

###### 3.3.2.2.2 Support from additional staff

Many M4M participants highlighted how Breathe staff played an important role in making the programme an **acceptable** and positive experience for them. Staff were repeatedly described as “*welcoming*”*, “warm*”*, “caring*”*, “attentive*” and “*supportive*”, as they took the initiative to provide mothers with snacks and drinks, as well as take care of their babies:

“If you needed to go to the toilet or get a drink you really felt comfortable in asking them to help you out with the baby.” (M3)

Music leads also emphasised that it was **feasible** to deliver M4M because they were supported by a project manager who was responsible for safeguarding and acting as “*a point of consistent contact for the mothers*”, alongside support officers who could provide additional practical and social support to mothers during and outside of the sessions:

“When you’ve got screaming twins that won’t stop crying, what’s the music lead supposed to do about that on their own whilst holding the session? You have to have that extra person in the room because especially with the group of women we’re working with, that’s a very stressful situation and so you need someone to be able to respond to that.” (S6)

This quote shows how the presence of dedicated support staff was key to ensuring participants' emotional safety even in moments of high stress and enabling music leads to focus on delivering session content without being overwhelmed by competing demands.

#### 3.3.3 Context

Three ingredients referred to the “context” of M4M—these are sub-categorised into the setting and project set-up.

##### 3.3.3.1 Setting

Two “context” ingredients referred to the setting of M4M (i.e., the circumstances and conditions making up the surrounding environment of the activity). These included location and transport, and the time and day of sessions.

###### 3.3.3.1.1 Location and transport

Opinions varied on whether the locations of M4M sessions were convenient and accessible. M4M participants said it was **feasible** for them to get to most sessions, as venues were either within walking distance or had good transport links. While some women found their journeys “*stressful*” or “*depressing*”, others enjoyed their commute:

“It was a bus ride for us to get to the venue, but I didn’t mind that…your world gets a bit small when you have a baby so it’s nice to get out and about.” (M11)

Although travel distance and cost did not deter participants from attending M4M, they recognised these factors could prevent other mothers from participating:

“I think for some mums, and I suppose on a longer-term basis, if there was a thing like this running, it was a little bit far for me. So having them a little bit more local.” (M4)

To overcome this potential barrier, participants and stakeholders suggested running M4M at a wider range of community venues, subsidising transport fees, and offering online sessions, although some raised concerns about whether an online programme would provide the same benefits:

“The music doesn’t work in the same way, the engagement isn’t the same, you don’t get the same experience of singing a harmony or a round with people, and you are still really only singing on your own at home and you’re not getting that group experience.” (S1)

###### 3.3.3.1.2 Time and day of sessions

Generally, M4M participants found it **feasible** to fit the programme around the routines of their baby and other children, but recognised that the time and day of sessions could be a barrier for other mothers:

“It wasn’t great timing actually because of nap times…my eldest goes to the nursery anyway. I guess if she hadn’t or it had been on one of the days that she doesn’t go to the nursery then that would’ve been an issue.” (M5)

Acknowledging that it was difficult to find a time that would work well for all families, participants and stakeholders recommended exploring different childminding options:

“A space where mothers can actually bring the other children as well, a protected space within the same environment, where other children can be looked after by nursery or professional staff.” (S12)

##### 3.3.3.2 Project set-up

The last “context” ingredient referred to how the project was set up and how participants were enrolled into the programme.

###### 3.3.3.2.1 Recruitment and advertising

M4M participants and stakeholders felt that M4M was advertised in an **acceptable** way. To make the programme more appealing to mothers, stakeholders described using marketing language around anxiety and low mood, or focusing on elements of fun and socialisation, rather than explicitly mentioning PND given the stigma potentially associated with it. Mothers also liked that recruitment information allayed any fears or hesitations they had about singing:

“It said something along the lines of “nobody will be forced to sing by themselves” and I found that so reassuring, so whoever put that on their website was really clever.” (M15)

Nonetheless, M4M participants and stakeholders felt that recruitment strategies could be improved. As many women found out about M4M through word-of-mouth and then registered online, they recognised these methods could have missed mothers who are more socially withdrawn or less tech-savvy. Breathe staff described unsuccessful attempts at engaging with social prescribing link workers, as mothers with PND tended to be under the care of a healthcare professional, while lack of time and buy-in also impeded referrals from clinicians. To strengthen partnerships with healthcare professionals, stakeholders recommended conducting taster sessions, sharing research evidence on M4M and communicating feedback on the progress of participants to their referrers:

“As a perinatal service you get a lot of emails about different groups and things they’re running. And as a busy clinician you don't always have it in mind…having had a positive experience of referring someone, it is a bit more at the front of my mind to offer to people.” (S7)

## 4 Discussion

In the context of the growing global burden of PND and limitations of traditional treatment pathways, art-based interventions offer a promising alternative for supporting maternal mental health and wellbeing (19, 20). This study evaluated the acceptability, appropriateness and feasibility of a group singing programme (M4M) for women with PND. High levels of acceptability, appropriateness, and feasibility were reported and insights were gathered into the core and peripheral ingredients of M4M that made the programme acceptable, appropriate, and feasible.

### 4.1 Core and peripheral ingredients

Several ingredients stood out as “core” to M4M based on the prominence and strength of these themes within the data from both mothers and professional stakeholders.

First, the **artistic content**, which involved ***singing a diverse range of songs in a group***, was acceptable and appropriate. This finding aligns with a scoping review of community music activities for wellbeing which found that using participant requested songs or that reflect their cultural origin helped facilitate engagement (39). Our findings extend this concept further by demonstrating that a combination of both new and familiar songs from *different* cultures are acceptable; while community music interventions for mothers in Iceland (40) and The Gambia (41) have utilised culture-specific repertoires, M4M's multicultural repertoire, enriched by inviting participants to introduce songs from their own countries and languages, was highlighted as a unique strength both in this study and a previous pilot RCT of M4M (42).

Second, the **social composition** of M4M, comprising ***a small group of mothers with shared mental health experiences and their babies***, was acceptable and appropriate. The importance of shared lived experience of mental health difficulties for creating a safe space has been similarly emphasised in a nature-based programme for mothers with PND (43) and a group dance programme for young people with anxiety (44). The inclusion of babies within sessions was also particularly suitable to support attachment for mothers with PND who may be struggling to connect with their infants, in line with existing research showing the relationship between early bonding and maternal mental health (45).

Third, the **activity facilitation** of M4M, which involved ***skilled and inclusive music leads supported by experienced and caring Breathe staff***, made the programme acceptable to participants and feasible for stakeholders to deliver. The presence of a music lead, project manager, and support officer(s), each with their separate roles and expertise, appears to be a distinctive feature of M4M (46) compared to other community music interventions for mothers that are delivered solely by musicians (40, 47).

The study also revealed ingredients that are potentially “peripheral” to M4M. It is striking that compared to the “project” and “people” aspects of M4M, the “context” of M4M was discussed with less emphasis and depth in participant narratives. While mothers and stakeholders described a relaxing, safe and comfortable atmosphere, they attributed this more to the people involved rather than the setting. It is possible that the Children & Family Centres may have contributed to the perception of a safe physical space, but this was not explicitly mentioned by mothers or stakeholders, indicating that it could be a modifiable aspect. A previous process evaluation of M4M suggested that other community venues (e.g., church halls) might be suitable, although size and comfort of the room were identified as important (46). An evaluation of the online adaptation of M4M also demonstrated that a virtual environment could be feasible and effective without diluting the impact of the programme on PND symptoms (26).

### 4.2 Implementation barriers and potential strategies

While feedback from participants and stakeholders was overwhelmingly positive, a small number of women did not find M4M acceptable, appropriate or feasible and several barriers to future implementation were identified. Participants mainly identified barriers to access such as **travel distance and costs**, and the **routines of their babies and other children** disrupting their schedules. Mothers and stakeholders suggested providing support for transport and childminding costs, which was enacted by the SHAPER team in subsequent sessions. Another proposed strategy was to offer M4M online for those unable to attend in person, making it a hybrid programme. However, there were concerns that the musical and social experience would be inferior, and technological barriers would be introduced. While a feasibility study of M4M delivered online during the COVID-19 pandemic found the adapted programme to be effective for reducing postnatal depression symptoms (26), there was no impact on loneliness and many participants expressed a preference for in-person sessions. Indeed, online delivery not only involves changes to the “context” but also to “people” and “project” ingredients, including limited opportunities to sing in harmonies and rounds, socialise outside of sessions, and receive hands-on support from staff (48).

Stakeholders mainly described **difficulties with recruitment and referral** of women due to lack of time, buy-in and knowing the most appropriate healthcare services to target, which mirror a prior evaluation of M4M (46). When scaling up M4M, participants and stakeholders emphasised the importance of partnering with professionals to refer women who may be harder to reach, rather than relying on self-referral. While creative health programmes targeted at other populations have successfully used social prescribing as a referral pathway (49, 50), our findings suggest that traditional social prescribing models might not be appropriate for women experiencing PND and that healthcare professionals might be better placed to refer women directly. However, mistrust towards healthcare professionals has been reported as a barrier to accessing maternal mental health services and support more widely, especially among women from minority ethnic backgrounds (43, 51). Community engagement could be an alternative tool to help mitigate medical mistrust (51), such as engaging mothers who have completed M4M as ambassadors for the programme. Ambassador training for people who have experienced stroke and taken part in a performance arts programme is being evaluated as part of the wider SHAPER project (52), and although M4M does engage programme ambassadors on an ad-hoc basis, the possibility of taking a more structured approach to M4M ambassador training is being explored. Forming an ethnically diverse network of ambassadors could enhance the credibility of M4M among women, allow mothers to stay connected after the programme and build stronger capacity for scale-up.

### 4.3 Implementation tensions and scaling considerations

In addition to the above barriers, some areas of implementation tension require further consideration.

Firstly, there is a need to balance the social and musical aspects of the programme. While communal singing itself can foster non-verbal bonding (42)—which could be especially valuable for mothers who find it difficult to articulate their emotions due to PND—providing an allocated space and additional time for informal interactions before and after sessions could further enhance social connection without compromising the musical focus during sessions. Attention should also be paid to the size of future M4M groups, ensuring that they are large enough for meaningful music-making, but small enough to foster trust and amplify the benefits of shared vulnerability, which is vital for mothers with PND who may be struggling with shame, guilt, or stigma (53). It is important to note that the smaller groups (>6 women) in this trial are not representative of the typical M4M group size (10–12 women) in community settings and reflected early challenges with recruitment to the study rather than to the intervention itself.

Secondly, it is crucial to balance the challenge and achievability of activities to facilitate a sense of accomplishment, bearing in mind the varying musical abilities that may exist within a group. This consideration has been highlighted in other creative health programmes (54, 55), but could be particularly pertinent to women with PND who may be experiencing difficulties with concentration or memory, and feelings of inadequacy. Additional support may be needed for those who struggle to engage musically (e.g., additional repetition of parts, reminders about where songs and lyrics can be accessed after the sessions) and additional stimulation could be provided for those with more musical experience (e.g., leading rounds, playing instruments).

Finally, high quality scale-up will require striking the right balance between flexibility and fidelity, allowing responsiveness to the diverse needs and circumstances of women with PND while ensuring that the “core” elements of M4M identified in this study are largely retained. In particular, the perceived centrality of the “people” ingredients of M4M points to the need for adequate funding, partnership, training, and resources to sustain wider roll-out of the programme without losing its “personal touch”. Although concerns were raised in this study about maintaining consistency and quality when rolling out M4M more widely, since this study was conducted, M4M has been successfully implemented outside of London through working with local delivery partners to support music leads with session delivery.

### 4.4 Strengths and limitations

A major strength of this study is the use of the INNATE framework to guide systematic and thorough identification of the active ingredients of M4M, which can facilitate replication and comparison with other creative health interventions. Another strength is the inclusion of both participant and stakeholder perspectives, which helped to identify common facilitators to implementation success from referral through to programme engagement, as well as some distinct barriers and areas of tension. In particular, the inclusion of divergent views, such as participants who found the programme more logistically challenging or less musically fulfilling, enriched the analysis by informing practical recommendations and potential implementation strategies. Additionally, conducting interviews a few weeks after programme completion struck a balance between minimising memory decay and allowing for meaningful reflection, as participants were able to recall specific details of the session (e.g., the repertoire) while also offering post-programme insights (e.g., ongoing social connections).

A limitation of this study is that only participants in the earlier sessions of M4M were interviewed, so we are missing the experiences of women and stakeholders as the project developed. As the few participants who scored low on the AIM, IAM, and FIM took part in later sessions, we lack insight into why their experiences of M4M were less positive. Moreover, online self-registration may have excluded mothers with lower digital literacy or limited internet access taking part in the trial, potentially introducing selection bias. Furthermore, the qualitative sub-sample included fewer women from ethnic minority groups and lower socio-economic backgrounds than the quantitative sample, and we did not speak to mothers who declined to participate or disengaged to understand their reasons. These women may have found M4M less acceptable, appropriate, and feasible than those who were interviewed, although findings from the quantitative survey did indicate high levels of acceptability, appropriateness and feasibility across the wider sample. Finally, the timing of interviews, which was shortly after the easing of COVID-19 restrictions, could have influenced our findings. For example, the isolation and loss of services experienced during the pandemic could explain why participants particularly valued the opportunity for social contact and support, although the social element was also identified as a key element of M4M in the original trial (42).

## 5 Conclusion

Overall, M4M was perceived as highly acceptable, appropriate and feasible to participants and stakeholders. Several “people” and “project” ingredients were identified as key facilitators to implementation success, while implementation barriers and strategies were identified regarding the broader “context” of M4M. Our study identifies the “core” ingredients of a creative health programme that leads to improved mental health for women experiencing PND. These findings can be used to inform the design of future creative health programmes targeted at improving mental health. Further evaluation could examine the mechanisms linking these ingredients to clinical outcomes and whether alterations to ingredients affects outcomes.

## Data Availability

The original contributions presented in the study are included in the article/Supplementary Material, further inquiries can be directed to the corresponding author.
