# Supplementary material for: Implementation of singing groups for postnatal depression: experiences of participants and professional stakeholders in the SHAPER-PND randomised controlled trial
Source: Front Health Serv. 2025 Jul 4;5:1582517. doi: 10.3389/frhs.2025.1582517 (PMC12271174; doi:10.3389/frhs.2025.1582517)
Supplement: Supplementary file 2 [file Table2.docx]

# **ADDITIONAL FILE 2:**

# Worksheet to identify the active ingredients of arts for health activities

**ACTIVE INGREDIENTS WORKSHEET**

The following worksheet is designed to help you to identify the active ingredients of your arts and cultural activity. Under each heading, you’ll find questions that ask you to reflect on different ingredients, with space to write in the ingredients relevant to your activity – we’ve listed examples in italics to show you where to write. Please note that not every ingredient will be present within your activity, so you can write ‘not applicable’ or leave blank the boxes which aren’t relevant.

**PART ONE: PROJECT**

| ATTRIBUTES | |
| --- | --- |
| 1. What is the format of your activity? | |
| Mode  Describe the form in which the activity occurs | *In-person.* |
| Synchroneity  Explain the degree to which the activity or engagement occurs in real-time for participants | *Live/real-time.* |
| Activity level  Tell us about the extent to which the activity requires active participation | *Active/participatory.* |
| Additional comments | *N/A* |
| 1. How much (i.e., what ‘dose’) of your activity is received by participants? | |
| Frequency  Explain how often the activity occurs | *Once a week.* |
| Duration  Describe the length of one dose/exposure of the activity in time | *1 hour.* |
| Maintenance  Tell us over what time periods the activity continues | *10-weeks.* |
| Additional comments | *N/A* |
| 1. What is the design (structural plan) of your activity? (This may or may not be adaptable.) | |
| Structure  Describe how the activity is organised in its delivery | *Semi-structured: unstructured socialising/welcome, warm-up which consisted of stretching, breathing and throat exercises, then into structured singing and time for unstructured socialising again at the end.* |
| Guiding  Explain whether the activity is taken in a particular direction to reach a goal or outcome | *Taught.* |
| Project approaches  Describe approaches or techniques employed within the activity that characterise it | *Participants copied the singing lead to learn the songs and then sang together, in harmony and in rounds.* |
| Personalisation  Explain whether the activity is designed to meet participants' needs or preferences | *A range of songs were used including upbeat, emotional, fun and calming songs to support the mental health of participants and to give participants a range of songs to use in different situations with their babies. Songs are used from different cultures in different languages for inclusivity.* |
| Challenge  Describe the intended level of difficulty of the activity, including whether the difficulty can be adapted across participants | *Easy and accessible. Some of the songs were catchy, memorable and include repeated phrases or melodies. Participants sang in harmonies and rounds.* |
| Goal orientation  Tell us about whether the activity is directed towards a particular aim or ends, including who sets goals | *Non goal focused.* |
| Feedback  Describe if and how evaluative information or reflections are included in the design of the activity | *Informal non-judgemental and positive feedback was provided to the group by the singing lead during the sessions.* |
| Additional comments | *N/A* |
| 1. What is the artistic content of your activity?   (Please note that artistic resources are included in the next question) | |
| Genre  Describe the primary branch of creative activity in which the activity(ies) is(are) categorised as well as any sub-categories | *Music and singing (familiar, popular and songs from different cultures in different languages).* |
| Multi-modality  Tell us if the activity also draws upon a range of different art forms or disciplines in its delivery | *N/A* |
| Activity type  The kind(s) of activity(ies) included that define(s) the creative engagement | *Group singing.* |
| Themes  Tell us if there is engagement with specific themes or subjects as part of the artistic content | *Singing leads could set their own repertoire which usually included a range of songs including positive and upbeat, inspiring, calming and emotional as well as songs from popular culture and songs from different cultures in different languages. Topics included songs about motherhood, and song lyrics were sometimes adapted to reflect experiences of motherhood. Songs were aimed predominantly at mothers rather than the babies, but some children’s songs and lullabies were included.* |
| Additional comments | *N/A* |
| 1. What resources (physical, conceptual or informational) materials are used/employed in the delivery of your activity? | |
| Activity consumables  List activity resources that can be used up or depleted | *None* |
| Props  List items such as objects or furnishings employed within the activity | *Selection of instruments including shakers and drums, cushions, blankets, chairs and mats. The singing lead sometimes played an instrument (e.g. ukulele).* |
| Products  List tangible outputs of the activity | *Recorded songs were available after the singing sessions as well as lyrics and signposting links to additional mental health support provided by charitable organisations, all via a password protected Breathe Melodies for Mums participant hub.* |
| Performances  List intangible outputs of the activity (incapable of being perceived by the sense of touch) | *N/A* |
| Additional comments | *N/A* |
| 1. Are there any activities that are integrated into the arts/cultural activity?   (NB. Collaborations or co-produced projects which form part of the project’s set-up shall be returned to in part three.) | |
| Psychosocial support  Describe any integrated professional techniques and/or resources designed to support mental health, wellbeing, experiences of psychological disorders and/or quality of life | *Stretching and breathing techniques were often used in warm-ups.* |
| Allied therapies  Describe any integrated therapeutic techniques that treat or manage physical disability, malfunction, pain or stress and tension via physical methods | *N/A* |
| Health education  Detail any integrated learning experiences designed to help individuals and communities improve their health by increasing their knowledge or influencing their attitudes | *The sessions did not include any formal health education, but the location of a Children & Family Centre contained posters and leaflets about maternal and infant health as well as access to specialist staff. The Breathe staff member supporting the session could provide 1:1 bespoke and geographically targeted signposting to services for participants if identified as requiring additional support or on request. There were one or two occasions where a health and wellbeing professional came in to talk about their services with the group at the end of the session, but this was not standard practice across all groups.* |
| Spiritual or holistic practice  Detail any integrated experiences relating to religious, spiritual or mind-body practices | *N/A* |
| Socially-engaged practice  Describe any integrated programmes designed to engage with social issues or that seek social or political change | *N/A* |
| Additional comments | *N/A* |
| ENGAGEMENT | |
| 1. Do you employ/use/engage with any objects, actions, materials or experiences that activate the senses as part of the activity (sensory stimuli)? | |
| Sight (Vision)  Explain if participants perceive objects or imagery by use of their eyes as part of the activity | *Sight was engaged (particularly eye contact) when singing in rounds.* |
| Hearing (Auditory)  Explain if participants perceive stimuli by ear as part of the activity | *Participants listened to the singing lead and to each other singing.* |
| Smell (Olfactory)  Explain if participants perceive odour or scent through the nose as part of the activity | *N/A* |
| Taste (Gustatory)  Explain if the act of tasting food or drink is part of the activity | *There was time to socialise and provision of snacks and drinks after the sessions.* |
| Touch (Tactile)  Explain if the body (i.e., hand, finger) is put in contact with something (i.e., an object or person) as part of the activity | *Participants held and interacted with their babies. Cushions and blankets to sit on and shakers were available.* |
| Additional comments | *N/A* |
| 1. Do you employ/use/engage with any objects, actions, materials or experiences that activate cognitive and/or creative processes as part of the activity (cognitive stimuli)? | |
| Involvement of the imagination  Explain if mental images or concepts beyond the senses are part of the activity | *Participants were sometimes encouraged to engage with the images of the songs and try their own harmonies. Participants were sometimes encouraged to suggest or share songs from their own cultural background or that they have grown up with.* |
| Emotional stimuli  Explain if there is something about the activity or aspects of it that brings about affective states of consciousness (feelings) | *A range of songs to stimulate different emotions. Positive music used to uplift participants, songs about motherhood which could prompt an emotional response, calm and gentle songs to relax participants and humorous songs to make participants laugh.* |
| Cognitive stimulation  Explain if there is something about the activity or aspects of it that prompts mental processes of perception, memory, judgment, and reasoning (contrasted with emotional processes) | *Participants learned lyrics of new songs and songs in different languages.* |
| Aesthetic engagement  Describe if participants engage with the activity through subjectively sensing something as beautiful | *Participants were encouraged to see the songs they sang and created as beautiful.* |
| Pleasure  Explain if there is something about the activity or aspects of it that brings about feelings of pleasure | *There were often moments of fun, joy and laughter during the singing sessions prompted by specific songs and informal chat and the singing lead entertaining the group.* |
| Participant choice  Describe if participants make selections from a number of possibilities based on preference or under guidance as part of the activity | *Participants were given some choice in the sessions, such as between two different songs to sing, and they were encouraged to bring songs from their own cultural background to the sessions.* |
| Additional comments | *N/A* |
| 1. Are any physical bodily motions or actions employed as part of the activity? | |
| Proprioception (or kinaesthesia)  Explain if heightened awareness of the body's position and movements is part of the activity, and whether it’s free or guided | *Participants became aware of their bodies through breathing, and stretching activities during the warm-up exercises.* |
| Movement  Tell us if bodily movement is prompted by the activity | *Stretching activities were used in the warm up at the start of the session.* |
| Physical exercises  Explain if any exercises are employed as part of the activity to promote bodily fitness and strength | *N/A* |
| Additional comments | *N/A* |

**PART TWO: PEOPLE**

| SOCIAL COMPOSITION | |
| --- | --- |
| 1. Who are the people involved in the activity (social diversity)? | |
| Presence of others  Detail the number of people present and/or the size of the group that the activity entails | *A group of between 3-13 people.* |
| Shared attributes  Describe whether individuals engaging together in the activity have characteristics in common | *All were mothers aged 18+ scoring 10 or more on the Edinburgh postnatal depression scale (EPDS) with babies aged between 0-9 months at the start of the programme.* |
| Distinct attributes  Describe the diversity of individuals engaging together in the activity | *There was some racial and socioeconomic diversity within the groups.* |
| Personal attributes  Describe any other personal qualities that participants have which inform how they engage with the activity | *N/A* |
| Additional comments | *N/A* |
| 1. Do participants have any previous experiences which are relevant to how they engage with the activity? | |
| Activity experience  State whether individuals engaging have previous experience(s) of the activity | *Several participants had previous experience of singing in choirs, while others had no formal group singing experience.* |
| Health experience  Outline whether individuals engaging have previous experience or knowledge of specific health conditions, healthcare in general, or health outcomes | *No previous experience of arts activities in healthcare contexts was required. An official diagnosis of postnatal depression was not required to participate in the programme.* |
| Lived experience  Tell us whether individuals engaging have personal experiences relevant to who the activity is tailored for | *All participants had some experience of low mood, stress or anxiety symptoms and most had recently accessed maternity healthcare.* |
| Relationship to others  Explain the kind of relationships the individuals engaging together in the activity have to one another | *Mothers brought their babies along to the group sessions.* |
| Additional comments | *N/A* |
| 1. Are there any social interactions (face to face or digital) part of or integrated into the activity? | |
| Shared focus  Explain if and how attention is given collectively to an object, activity, thought or person/people as part of the activity | *Joint focus on the singing leader during the sessions.* |
| Shared activity  Explain if and how participants cooperate or collaborate as part of the activity | *The full group sang together with the singing leader. Songs sometimes required rounds or harmonies, call and response.* |
| Social exchanges  Describe the social elements of the activity itself that involve interaction with others | *Participants were given time to chat at the beginning and end of the session with one another and the singing lead.* |
| Structured social time during activity  Describe any aspects of the activity that encourage the formation of social relationships or socialising | *Facilitated introductions and welcome at the first session and time to chat at the beginning and end of each session.* *The Breathe staff member set up a WhatsApp group for participants to form social relationships during the programme.* |
| Structured social time outside of activity  Detail any planned time outside of the core activity delivery used to encourage the formation of social relationships or socialising | *N/A* |
| Communications  Tell us if and how those in leadership/management communicate with participants in the lead up to and after the activity | *Participants were sent an email or text with a welcome pack and the details on how to join the session by the supporting Breathe staff member. ‘Check in’ emails and calls were also made to participants to see how they were doing or follow-up on absences. Participants received weekly text reminders to attend sessions. Access to the Breathe Melodies for Mums participant hub was shared by the supporting Breathe staff member. Participants did not have direct contact from the singing lead.* |
| Additional comments | *N/A* |
| 1. Are there social exchanges (face to face or digital) that that are not planned as part of the activity | |
| Unstructured social time during activity  Describe if space is provided during an activity for participants to informally socialise | *At the beginning and end of sessions participants had a few minutes to say hello and chat to the others in the class.* |
| Unstructured social time outside of activity  Detail if social time or social activities are shared between participants outside of the activity, without formal guidance | *Participants would often go for a coffee after the sessions or proactively arranged to meet outside of the sessions. Participants could communicate with one another via a WhatsApp group if they had opted in. WhatsApp groups were moderated by a Breathe administrator. Upon joining, the Breathe administrator shared WhatsApp group guidelines explaining how the groups were intended to be a welcoming and safe space, focused on the shared experience of participating in Breathe’s programmes and other activities that promote wellbeing. Principles included being kind and courteous, sharing wisely, respecting everyone’s privacy, and sharing concerns with the administrator. Failure to adhere to these guidelines may result in being removed from the groups. Participants were notified that the Breathe administrator would exit the group 2 months after sessions have ended, and if participants chose to remain in the group, then Breathe would take no responsibility for any of the messages or content shared. Frequency of communication was led by the participants.* |
| Additional comments | *N/A* |
| ACTIVITY FACILTATION | |
| 1. What kind of facilitation (i.e., the people who lead, guide or facilitate the participant-facing aspects of the activity and not the administrative aspects) is employed? | |
| Facilitator(s)  Explain who facilitates the activity during its delivery and what form this facilitation takes | *A singing lead facilitated the sessions, and a Breathe staff member provided practical/social support to participants and their babies.* |
| Co-production  Describe if the activity involves actively including participants in the process of delivering and facilitating it | *Participants were invited to bring some song suggestions to the classes and help in the creation of group songs.* |
| Number  Detail the number of people who facilitate or lead the activity | *One singing lead and one Breathe staff member, occasionally joined by volunteers and/or student placements.* |
| Professionalisation  Tell us about whether the person/people who facilitate the activity identify as professional(s) within their specific field/domain | *Professional singers who may perform professionally and run other singing activities, especially in arts and health.* |
| Training  Explain if the person/people who facilitate the activity have professional training (i.e., domain-specific skills) | *Singing leads were supported with regularly briefing, debriefing and training opportunities delivered by Breathe focussed on their delivery/practice but within the context of working with participants with PND. The supporting Breathe staff member was experienced in working with vulnerable people and was trained in safeguarding.* |
| Consistency  Explain whether facilitation changes or stays the same | *There were a number of singing leads but one singing lead and one Breathe staff member was assigned to work with the same group for the entire 10-weeks. Replacements would happen if someone was away or unwell.* |
| Additional comments | *Both singing leads and Breathe staff were supported by an Employee Assistance Programme providing confidential, generalised support and advice on all areas of their lives.* |
| 1. If there is a facilitator, what experience do they bring to the delivery of the project? | |
| Activity experience  Tell us about the amount of previous domain-specific experience, knowledge or skills the facilitator(s) have | *The singing leads had past experience in leading similar workshops for mums and babies as well as extensive community music leadership.* |
| Health experience  Outline the amount of previous experience or knowledge the facilitator(s) have in relation to specific health conditions, healthcare in general, or health outcomes | *The singing leads were specialist arts and health leads, although not necessarily working with PND before the project.* |
| Lived experience  Tell us if the facilitator(s) have personal experiences relevant to who the activity is tailored for | *Some of the singing leads had experience of being a mother.* |
| Relationship to others  Tell us if the facilitator(s) have any pre-existing relational experiences of engaging with participants | *The singing leads didn’t know anyone in the group before the sessions began.* |
| Additional comments | *N/A* |
| 1. In what style/manner is the activity is delivered and what artistic practice is drawn upon? | |
| Technique  Explain whether the activity facilitator(s) draw on approaches or technical skills that are characteristic of one’s domain-specific field | *Vocal pedagogy (singing lead).* |
| Personal attributes  Detail any additional qualities that the facilitator(s) bring to the activity that informs how it is delivered | *The singing leaders had excellent communication skills and were good listeners. They were enthusiastic and fun. The supporting Breathe staff member was empathetic and intuitive to participants requiring additional support to engage.* |
| Values-directed focus  Explain if and how the facilitator(s) deliver the activity based on specific ethical values | *The singing leads sought to be inclusive by fostering a space where all were able to ask questions, where there was no ‘right’ way to sing or to engage. They respected participants and sought to be responsive to participant needs. Mothers were encouraged to accept all potential disruptions from their babies without judgement and to participate or watch as required to fit in with feeding or changing or comforting of their infants.* |
| Outcomes-directed focus  Explain if and how the facilitator(s) focus on health, educational, or aesthetic goals as part of the activity | *The focus was on learning songs to support mental health, improve confidence interacting with their babies and bonding with their babies.* |
| Person-centred focus  Explain if and how the facilitator(s) consider participant preferences, needs, and values to deliver the activity | *The singing lead and Breathe staff member were friendly, warm and welcoming and made an effort to speak to every individual participant, so they felt welcomed. Participants were asked if they had any particular needs during and in between the sessions. The Breathe staff member offered to hold the baby or watch the baby if the participant needed a break.* |
| Autonomy-directed focus  Explain if and how the facilitator(s) provide participants with autonomy as part of the activity | *Participants were given some autonomy in how they engaged (e.g. which instruments to use), how to move their babies, and some selection over songs.* |
| Equality, Diversity and Inclusion  Explain if and how the facilitator(s) consider fair treatment and equal opportunities to deliver the activity | *The singing lead actively chose songs in different languages and from different cultures, sharing origin, meaning and context, and encouraged mothers to bring their own songs to the classes too. Song lyrics were considered carefully for inclusivity. On registration, participants were asked whether they had any access needs. An access budget was included in the programme budget should participants require support to join such as travel expenses.* |
| Safety  Explain if and how the facilitator(s) consider the safety of participants in how the activity is delivered | *The supporting Breathe staff member was trained in safeguarding, had previous experience of working with vulnerable people and was able to respond to any concerns for participants in the group. The singing lead and Breathe staff member wore Breathe t-shirts so they were clearly identifiable as leading the sessions. Risk assessments are conducted for each venue. Singing lead and Breathe staff, volunteers and/or placement students all completed enhanced DBS checks and references.* |
| Tailoring  Explain if and how the facilitator(s) personalise or adapt the activity to meet the needs of participants | *The singing lead provided different ways for mothers to engage depending on their level of confidence e.g. adding harmonies or simple instruments, participating in rounds.* |
| Additional comments | *N/A* |
| 1. Are there additional staff or other people that support, co-lead or are present at the activity? | |
| Presence of volunteers  List any unpaid staff who support or co-lead the delivery of the activity and explain what they do to support | *Occasionally, placement students and/or volunteers with Breathe Arts Health Research supported with administration of sessions and shadowed delivery.* |
| Presence of healthcare professionals  List any healthcare professionals and how they support or co-lead the delivery of the activity | *Healthcare professionals were not present in the workshops, but there was a safeguarding protocol in place for any concerns to be addressed and escalated if required.* |
| Presence of others  Detail whether there are any other staff or people and how they support, co-lead or are present for the delivery of the activity | *A Breathe staff member always attended the groups to provide practical and social support and support with the babies. This allowed the singing lead to focus on creative delivery.* |
| Additional comments | *N/A* |

**PART THREE: CONTEXTS**

| SETTING | |
| --- | --- |
| 1. What circumstances, objects, and conditions make up the surrounding environment of the activity? | |
| Location  Describe the place where the activity is delivered | *Indoors at a Children & Family Centre.* |
| Basic features  Describe the functional aspects of where the activity takes place and how the room(s)/space(s) are arranged | *The venues were warm and well lit. There were good basic facilities. Soft blankets, cushions and mats were placed on the floor for mothers and babies to sit on. Chairs were also available for participants to sit on. Toys and shakers were available for the babies to play with. Participants tended to form a semi-circle with the singing lead at the front.* |
| Attractiveness  Explain whether the environment of the activity is perceived as beautiful, attractive or pleasing to the eye. Note any modifications made. | *The environment is colourful, comfortable and informal with the focus on children and babies within Children & Family Centres.* |
| Situation  Outline the geographic and/or socioeconomic features of where the activity takes place | *The singing groups took place in South East London. Some of the children’s centres were in deprived areas.* |
| Time and day  Detail when the activity takes place and if this changes | *Singing groups took place at different times of the day and different days of the week for different groups, however the day and time was consistent for each group. The groups did however always take place between 10am and 4pm and on a weekday (not weekend).* |
| Access  Explain the methods used and the means or opportunities available to find and participate in an activity | *Most participants travelled between 5 to 90 minutes by either walking, driving or using public transport to get to the groups.* |
| Privacy  Tell us if the location of the activity is accessible by anyone (including those not engaging) or if it is only open to those who are part of the activity | *Private room in a Children & Family Centre.* |
| Additional comments | *N/A* |
| 1. What is the atmosphere (character, feeling, or mood) like where the activity takes place? | |
| Comfort  Explain the degree to which the setting of the activity elicits a sense of ease, safety and relaxation | *The play mats, cushions and instruments supported a comforting mood. The Children & Family Centre location helped participant’s feel safe and relaxed as well as opening up access to specialist services based there should they be required.* |
| Belonging  Explain the degree to which the setting and environment elicits a feeling of being included | *Every effort was made to ensure all felt welcome and comfortable to participate. Participants were encouraged to all sit /stand together on the play mats and chairs in the centre of the room. There was an optional WhatsApp group to encourage socialising outside of the groups and participants were given password protected access to the Breathe Melodies for Mums participant hub.* |
| Familiarity  Explain the degree to which the setting of the activity is known or unknown by participants | *Many people were fairly local to the Children & Family Centre. Some people did travel further and it was outside of their local community (sessions were open to women across London).* |
| Ambiance  Describe the mood or tone of the surroundings where the activity takes place | *The atmosphere was positive, welcoming, calm but also fun.* |
| Organisation  Explain how the delivery and management of the activity is perceived by participants | *Well organised with reminder texts and emails about classes each week and clear signposting and welcoming at the venue.* |
| Additional comments | *N/A* |
| PROJECT SET-UP | |
| 1. What economic resources (if any) are connected to the activity and its delivery? | |
| Participant charges  Describe any monetary transactions connected to or part of the activity, and if there are any support structures in place to ensure equal distribution of monetary resources | *It was free for participants to attend.* |
| Project funding  Explain if and how the activity is delivered using monetary resources obtained through external organisations, individuals, trusts, charities or the government to be used for the purpose of delivering the activity | *Funded for the purposes of the research study by a research grant.* |
| Fees  Describe if any people involved in the delivery of the activity are paid for their time | *All delivery staff were paid for their time.* |
| Longevity  Describe the duration of the activity across time and whether the activity can be upheld and supported with the economic resources available | *The intervention was run for the research study. It has since been taken on in a similar form through healthcare, statutory, private foundation and grant funding.* |
| Environmental sustainability  Tell us if best use of resources are made in view of what may be harmful to the environment | *There were no lyric sheets or other disposable resources.* |
| Additional comments | *N/A* |
| 1. What person, people, group(s) and/or company(ies) is/are in charge of organising the management of the activity? | |
| People  Describe if there are any people ‘behind the scenes’ of the participant-facing aspects of the activity | *The classes were supported behind the scenes by Breathe staff, researchers as well as the musicians involved with running the classes.* |
| Affiliation  Explain if and how the activity is connected to an organisation or institution | *Led by Breathe Arts Health Research.* |
| Branding  Describe the language, visual imagery, ethos and/or symbols that represent any connected organisations, institutions and/or the activities | *The logo for the organisation delivering the singing sessions combined an artistic and scientific aesthetic depicting a doodled brain using different shades of green. The language was welcoming, focussed on symptoms rather than diagnosis, and promoted emotional and mental wellbeing whilst also linking the impact of the activities to evidence and research.* |
| Collaboration  Describe any partnerships with other organisations involved in the delivery of the activity | *N/A* |
| Patient and Public Involvement  Describe if the activity involves actively including participants or the public in the process of designing and organising the activity | *The programme was developed and refined together with artists, researchers, and women experiencing postnatal depression.* |
| Additional comments | *N/A* |
| 1. How do participants find out about or become enrolled into the activity? | |
| Formal referral  Explain if there is a referral into the activity from professional services such as via established organisations or schemes | *Self-referral however healthcare professionals could signpost people to the group.* |
| Informal referral  Explain if there is a referral into the activity from personal, social group, or community connections or networks | *Friends and relatives could signpost women to the groups. Information was shared via social media.* |
| Choice  Tell us who decides if the participant will enrol in the activity | *Participant choice based on meeting eligibility criteria.* |
| Advertising  Describe how participants find out about the activity via publicity materials, and whether these materials are targeted for particular groups | *Flyers and posters advertised within baby weighing clinics, Children & Family Centres, libraries and shops, advertising via social media, word of mouth, and sending out information to healthcare professionals for signposting, and GP practice mail-outs coordinated by the National Institute for Health Research (NIHR) Clinical Research Network (CRN).* |
| Additional comments | *N/A* |
| 1. Do you signpost or refer to any services, resources, support, or advice beyond the activity itself? | |
| Inter-sector signposting  Describe any resources and information provided about other arts or cultural activities that may be suitable for the participant(s), or if participants are directly recruited into such activities | *Participants often shared their experiences of other arts/cultural activities with others in the group, either through structured or unstructured conversation. At the end of the course, participants were given information via email about other classes and arts/cultural activities in the area they could consider joining.* |
| Health-sector signposting  Describe any resources and information provided about healthcare or support for mental or physical health, or if participants are directly recruited into such activities | *The Breathe Melodies for Mums participant hub included signposting links to additional mental health support provided by charitable organisations and the NHS, and a signposting sheet with links to these services was provided at the end of the programme. The Breathe staff member supporting the session could provide 1:1 bespoke and geographically targeted signposting to services for participants if identified as requiring additional support or on request.*  *The venue the classes took place in had further resources and leaflets on maternal and infant health as well as specialist services.* |
| Social signposting  Describe any resources and information provided about social support services/activities, or if participants are directly recruited into such activities | *N/A* |
| Other-sector signposting  Describe any resources and information provided about other sectors, or direct recruitment into such activities | *N/A* |
| Safeguarding referral  Describe if and how action will be taken to protect participants from emotional or physical harm if it is needed | *If there was any concern over the welfare of mothers or their babies, either through observation at the classes or results on the questionnaires they completed as part of the research, they were contacted by the research team to discuss their wellbeing, and safeguarding protocols followed if necessary.* |
| Additional comments | *N/A* |

**FURTHER COMMENTS**

| Use the space below to note any further comments in relation to identifying the active ingredients of your arts or cultural activity. |
| --- |
| *N/A* |
