# Supplementary material for: Implementation of singing groups for postnatal depression: experiences of participants and professional stakeholders in the SHAPER-PND randomised controlled trial
Source: Front Health Serv. 2025 Jul 4;5:1582517. doi: 10.3389/frhs.2025.1582517 (PMC12271174; doi:10.3389/frhs.2025.1582517)
Supplement: Supplementary file 3 [file Table3.docx]

**Additional File 4. Topic guides for participants and professional stakeholders**

**Implementation interview topic guide for participants**

**I. Opening** [establishing rapport, providing purpose of the interview, seeking permission to audio record]

**II. Reasons for accepting to take part in the intervention**

1. Reasons behind deciding to take part in the intervention, and committing to attendance/completion
2. Factors participant hoped to get out of the intervention and whether this was met (expectations)
3. Any other interventions considered when making the decision on whether to take part
4. Anything else in relation to the decision to take part in the intervention

**III. Acceptability, appropriateness and feasibility of the intervention**

1. How acceptable/satisfactory was the intervention for the management of postnatal depression
2. How appropriate/relevant was the intervention in relation to postnatal depression
3. How feasible was attending/completing the intervention [how easy or difficult it was]
4. Factors that:
   1. made participant feel able/willing to complete/attend the intervention
   2. made it more difficult to complete/attend the intervention

**IV. Fidelity of receipt:** engaging, understanding, acquiring/learning and applying skills/knowledge taught

Exploring views and experience of the participant in relation to the content of the intervention - what participant has taken away from it, and the impact it has had on their [health condition].

1. Ability to engage with the content of intervention
2. Ability to follow/understand the content of intervention
3. Adherence to homework or practical tasks [if any; on a percentage scale, 0-100% of the time]
4. Skills/knowledge learned in the intervention and feasibility/ability of applying them day to day

**V. Intention to adopt and sustained use of learned skills/knowledge**

1. Intention to adopt/use learned skills/knowledge in everyday management of [health condition] [on a percentage scale, 0-100% of the time]
2. Ability to continue putting into practice learned skills/knowledge, going forward

[on a percentage scale, 0-100% of the time]; and:

- 1. Facilitators to sustained use of learned skills/knowledge, for the long-term, and
  2. Barriers to sustained use of learned skills/knowledge, for the long-term

**VI. Unintended consequences of your course**

1. Impact of the intervention on the participant and people around them, including:
   1. positive effects, and
   2. negative consequences
2. Willingness to recommend the intervention to others (incl. what could have been done differently]

**VI. Closing**

1. Anything else not covered in the interview that participant would like to add

**Thank you** very much for your help and taking the time to complete the interview.

**Implementation interview topic guide for professional stakeholders involved in M4M**

**[for deliverers and supporters: artists, clinicians]**

**I. Opening**

[*establish rapport*] I would like to ask you some questions about M4Ms in order to learn more about your experiences. We hope to use this information to help inform us on any ways that M4Ms could be improved in the future. The interview should take about 1h, and with your permission, I would like to audio record the interview. Would this be okay? Do you have any questions before we begin?

**II. Acceptability, appropriateness and feasibility of the M4M’s content (what it was delivered)**

Let me begin by asking you some questions about how acceptable, appropriate/relevant and feasible M4Ms is. Think about it in relation to the content and desired outcomes, in particular, the knowledge and skills taught to patients to help improve their PND symptoms and quality of life.

1. How acceptable/satisfactory/enjoyable did you find the programme content to deliver? Why?

- Prompt for artists: Are you happy delivering M4Ms in this way?
- Prompt for clinicians: Is this type of singing programme acceptable for people with PND?

[Definitions: *acceptability* = extent to which intervention is perceived as agreeable and satisfactory/enjoyable; *acceptable* = capable or worthy of being accepted or received with pleasure; pleasing to a receiver]

1. How appropriate/suitable/fit for purpose is the content of M4Ms for improving symptoms and quality of life in people with PND? Why? Prompt: Is this the right content to deliver?

[Definitions: *appropriateness* = extent to which intervention is perceived fit and relevant; *appropriate* = set apart/reserved for a particular use or person; suitable, fit and proper]

- [artists only] How feasible/doable is the content of M4Ms to deliver? Why? Prompt question: Can you deliver the content well? What might be the challenges or advantages in doing it?

[Definitions: *feasibility* = the extent to which intervention can be successfully used or carried out. *feasible* = possible or practical to do easily or conveniently.]

1. [context] Have you encountered any barriers in delivering the content of M4Ms? If yes: What would you say are the main:
   - barriers to delivering the content of M4Ms?
   - facilitators to delivering the content of M4Ms?

**III. Acceptability, appropriateness and feasibility of the programme structure (how it was delivered)**

We’ve talked about the content of M4Ms, let’s move on now to thinking about the programme in relation to how it was structured and delivered, the design and intensity, including the time needed to deliver each session, as well as any preparation you had to do within your role, including training.

1. How acceptable/satisfactory/enjoyable is the way in which M4Ms is structured and delivered? Why? Prompt: Are you happy delivering the programme in this way (i.e., one day a week over 10 weeks)?
2. How appropriate/suitable/fit for purpose is the way that M4Ms is structured and delivered (for improving symptoms and quality of life in people with PND)? Why? Prompt: Is this a good way to deliver the programme?
3. [artists only] How feasible/doable is it to deliver M4Ms in this way? Why? Prompt: Can you deliver the programme properly in this way?
4. Have you encountered any barriers in delivering the content of M4Ms? If yes: What would you say are the main:

- barriers to delivering the programme in this way? Prompt questions: Were there any challenges to you (as an artist)? How did you overcome them?
- facilitators to delivering the programme in this way?

**IV. Unintended consequences of the programme**

M4Ms is intended to help improve the symptoms and quality of life for people with PND. I would like to explore any unintended consequences that the programme might have had that are relevant to you, your team, mums and their care. These can be both positive and negative, including, workload, workflow changes, relationships with others in your team, negative/positive emotions or frustrations.

1. Did you experience any:

- negative consequences as result of M4Ms? How would you describe them? Prompt: in relation to yourself, your team, patients, and/or their carers/family.
- positive effects as a result of M4Ms? How would you describe them? Prompt: in relation to yourself, your team, patients, and/or their carers/family.

1. [artists only] Did the way in which M4Ms was originally planned to be delivered change? / Did you have to adapt the delivery to fit the participants and/or your setting?

- **If yes:** What were the adaptations?
- What are the reasons behind adaptations?
- How much time did adaptation add to your role in the programme delivery/implementation?

**V. Sustained use of M4Ms for management of PND symptoms and quality of life**

1. How willing would you be to adopt M4Ms (i.e. continue to support) and assist in the delivery again in the future? Why?
2. Going forward, how sustainable, in your opinion, is the delivery of M4Ms? Why? Prompt: How easy would it be to continue running M4Ms?
3. If M4Ms was rolled out in practice (i.e. made more widely available), what would you anticipate would be the main:

- barriers to sustained use and delivery?
- facilitators to sustained use and delivery?

1. On reflection, is there anything that you would change / could have been done differently to make M4Ms more appealing (to health care professionals and patients)? What might that be? Prompt: What could be done differently? What was done well?

**VI. Implementation strategies**

We would like to identify implementation strategies that have been used within your setting in order to enhance the delivery and implementation of M4Ms. Implementation strategies refer to methods or techniques used (within your setting) to enhance and promote the adoption, implementation, and sustainability of the programme. For example, building champions/ambassadors, promoting/increasing awareness of M4Ms amongst staff and patients and providing training to staff (note to interviewer: refer to the implementation strategies checklist for more detailed examples).

1. [artists only] What implementation strategies have you used within your setting during the trial?

[clinicians only] How did you identify and refer patient to M4Ms? How did you raise it with the patient? Prompts: What words did you use? Can you give me an example of how you approached the patient?

1. [artists only] What implementation strategies do you feel were effective/important and would recommend to centres wishing to implement M4Ms in the future (after the trial is complete)? /

[clinicians only] What worked?

1. [artists only] What implementation strategies do you feel were ineffective?

[clinicians only] What didn’t work?

**VII. Closing**

I appreciate the time you took for this interview. Is there anything else that we have not covered that you would like to add?

Thank you for your time and input.

**Implementation interview topic guide for potential referrers into M4Ms**

**I. Opening** [establishing rapport and purpose of the interview, seeking permission to audio record]. I would like to ask you some questions about M4Ms. The interview should take about 20-30min. and with your permission, I would like to audio record the interview. Would this be okay? Do you have any questions before we begin?

**II. Views/attitudes about M4Ms**

1. What do you understand about the M4M groups? (prompt format, structure, content, anything about the organisation delivering it).
2. What is your view about using a singing programme like M4Ms as a therapeutic option to help with the symptoms of PND?
3. How willing would you be to adopt a programme like this in the future and to refer other mums? Why?
4. Going forward, how sustainable would a programme like M4Ms be, in your opinion? Why?
5. What are your views on the referral pathway for individuals wishing to enrol in M4Ms? Could this be improved?
6. What would you say would be the potential:

- Barriers to sustained use and availability of such programmes?
- Enablers to sustained use and availability of such programmes*?*

**III. Closing**

Is there anything else not covered in the interview that you would like to add?

**Thank you** very much for your help and taking the time to complete the interview.
