# Supplementary material for: Implementation of singing groups for postnatal depression: experiences of participants and professional stakeholders in the SHAPER-PND randomised controlled trial
Source: Front Health Serv. 2025 Jul 4;5:1582517. doi: 10.3389/frhs.2025.1582517 (PMC12271174; doi:10.3389/frhs.2025.1582517)
Supplement: Supplementary file 4 [file Table4.docx]

**Additional File 4. Consolidated criteria for reporting qualitative studies (COREQ): 32-item checklist**

| **No. Item** | **Guide questions/description** | **Reporting** |
| --- | --- | --- |
| **Domain 1: Research team and reﬂexivity** |  |  |
| *Personal Characteristics* |  |  |
| 1. Interviewer/facilitator | Which author/s conducted the interview or focus group? | RD, MBL |
| 2. Credentials | What were the researcher’s credentials? E.g. PhD, MD | RD: PhD, MSc, BSc  MBL: MSc, BSc |
| 3. Occupation | What was their occupation at the time of the study? | RD: research scientist with background in health psychology and implementation science  MBL: medical student and freelance neuropsychology researcher |
| 4. Gender | Was the researcher male or female? | RD: Female  MBL: Female |
| 5. Experience and training | What experience or training did the researcher have? | RD: experience conducting qualitative interviews across patient and professional stakeholder groups  MBL: experience working as a research assistant in a range of studies |
| *Relationship with participants* |  |  |
| 6. Relationship established | Was a relationship established prior to study commencement? | No |
| 7. Participant knowledge of the interviewer | What did the participants know about the researcher? e.g. personal goals, reasons for doing the research | RD and ML explained to participants that the interviews were part of a wider research study and aimed to explore their experiences of the programme to inform future improvement |
| 8. Interviewer characteristics | What characteristics were reported about the interviewer/facilitator? e.g. Bias, assumptions, reasons and interests in the research topic | RD: Being a mother increased interest in research topic  ML: None reported |
| **Domain 2: study design** |  |  |
| *Theoretical framework* |  |  |
| 9. Methodological orientation and Theory | What methodological orientation was stated to underpin the study? e.g. grounded theory, discourse analysis, ethnography, phenomenology, content analysis | Framework analysis (Page 9) |
| *Participant selection* |  |  |
| 10. Sampling | How were participants selected? e.g. purposive, convenience, consecutive, snowball | Purposive (Page 7) |
| 11. Method of approach | How were participants approached? e.g. face-to-face, telephone, mail, email | Email, posters, via clinicians, social media (Page 7) |
| 12. Sample size | How many participants were in the study? | 37 in the qualitative work (22 mums and 15 professional stakeholders) and 109 participants completed the quantitative measures (Page 10) |
| 13. Non-participation | How many people refused to participate or dropped out? Reasons? | Of the 133 intervention participants, 109 participated in the quantitative implementation surveys. Two withdrew from the study (without providing reasons), 1 did not attend any M4M sessions and 21 did not complete the surveys for unknown reasons.  Of the 42 women approached for qualitative interviews, 13 did not respond, 2 did not want to be recorded, 1 was out of the country, 1 did not attend any M4M sessions, and 3 declined or did not turn up for the interview (Page 10). |
| *Setting* |  |  |
| 14. Setting of data collection | Where was the data collected? e.g. home, clinic, workplace | Via Zoom (Page 8) |
| 15. Presence of non-participants | Was anyone else present besides the participants and researchers? | Babies were often present with their mothers during the interviews (Page 8) |
| 16. Description of sample | What are the important characteristics of the sample? e.g. demographic data, date | Page 10 |
| *Data collection* |  |  |
| 17. Interview guide | Were questions, prompts, guides provided by the authors? Was it pilot tested? | Topic guides (Additional File 3) were piloted in previous trials (Page 8) |
| 18. Repeat interviews | Were repeat interviews carried out? If yes, how many? | No |
| 19. Audio/visual recording | Did the research use audio or visual recording to collect the data? | Audio and visual recording (Page 8) |
| 20. Field notes | Were ﬁeld notes made during and/or after the interview or focus group? | No |
| 21. Duration | What was the duration of the inter views or focus group? | 12-56 minutes, averaging 25 minutes (Page 8) |
| 22. Data saturation | Was data saturation discussed? | Yes (Page 8) |
| 23. Transcripts returned | Were transcripts returned to participants for comment and/or correction? | No |
| **Domain 3: analysis and ﬁndings** |  |  |
| *Data analysis* |  |  |
| 24. Number of data coders | How many data coders coded the data? | 3 (page 9) |
| 25. Description of the coding tree | Did authors provide a description of the coding tree? | Yes (Pages 9, 12-13) |
| 26. Derivation of themes | Were themes identiﬁed in advance or derived from the data? | Both (Page 9) |
| 27. Software | What software, if applicable, was used to manage the data? | NVivo (Page 9) |
| 28. Participant checking | Did participants provide feedback on the ﬁndings? | No |
| *Reporting* |  |  |
| 29. Quotations presented | Were participant quotations presented to illustrate the themes/ﬁndings? Was each quotation identiﬁed? e.g. participant number | Yes (Pages 14-24) |
| 30. Data and ﬁndings consistent | Was there consistency between the data presented and the ﬁndings? | Yes (Pages 14-24) |
| 31. Clarity of major themes | Were major themes clearly presented in the ﬁndings? | Yes (Pages 14-24) |
| 32. Clarity of minor themes | Is there a description of diverse cases or discussion of minor themes? | Yes (Pages 14-24) |
