# Supplementary material for: Implementation of singing groups for postnatal depression: experiences of participants and professional stakeholders in the SHAPER-PND randomised controlled trial
Source: Front Health Serv. 2025 Jul 4;5:1582517. doi: 10.3389/frhs.2025.1582517 (PMC12271174; doi:10.3389/frhs.2025.1582517)
Supplement: Supplementary file 5 [file Table5.docx]

**Additional File 5. Table of categories, ingredients and supporting quotes**

| **Category** | **Ingredient** | **Quotes from participant** | **Quotes from stakeholder** |
| --- | --- | --- | --- |
| **Project** | Frequency of sessions | “I think having something regular that I went to every week and that helped me build my confidence as well.” (M1)  “Having something in the calendar that I knew, every Tuesday I’d be going to Melody for Mums was positive because it afforded me a little bit more structure to my day outside of just feeding and eating.” (M14)  “But quite often it’s one of those sort of self-defeating things that when you’re in a particularly bad place you don’t think of things like that… So having something that's in the diary that you do go to every week I thought was quite helpful to kind of pull you out of that.” (M21)  “It was just kind of like it was a bright spot to look forward to in the week and it kind of helped give the week structure.” (M22) | N/A (participant theme only) |
|  | Length and number of sessions | “I think that was probably about right for a sort of term. Because actually looking back on it, that was almost three months of her, I felt like I was in a totally different place with her by the end of it as well.” (M1)  “I think it was the right amount of time. It wasn’t too long, it wasn’t too short. It just gives you enough time to settle and then do what you need to do in the session.” (M2)  “I mean it would be lovely if there a group like this just going on that you could just join. But yeah. But in terms of what you're trying to achieve, I think ten was good.” (M4)  “I think it is good that it’s a ten-week course... it makes it quite special and it sort of makes you go because you’re like, “I’ve only got ten of these so I don’t want to miss it,” it would be amazing if it fed into some sort of other, like gradually into kind of ongoing singing group that you can just drop in and out of.... I wouldn’t want any shorter than ten...Ten feels like it’s a good amount and it’s not scary to commit to.” (M11)  “Ten felt about right. I’ve done like stuff before where it’s been six weeks, but I think ten’s nice because you need that little bit extra time to get to know people so if you have missed a session for whatever reason, I think that you’ve got a higher probability of making sure you’re still getting a strong connection with people.” (M18) | “I think ten weeks feels right. The programmes that I’ve been through for ten weeks you really can see a difference and like the way people leave the room at the end all kind of bonded in together and they’ll like go for a coffee and… they always say, well, that went really quickly but actually then they’re always like, you know, wow, look how grown up our babies are… I wish we could just carry this on forever for these people, you know, they all want to carry on but there’s so many people in need… as a member of staff you have to kind of let go a little bit and be like right, we really made a big impact on these people, we’ll give them some signposting and tools to continue in the community but now onto the next group.” (S6)  “I’ve never thought of it being longer because I think we see that change in those symptoms in that 10-week period so it feels like the right time to not kind of continue. But anything shorter I think would be, you know, the number of weeks a mum might then be there is very small, too small so I do feel like 10 weeks is the right amount.” (S8)  “I guess it depends on commitments and everything, but maybe longer in a sense because you really see a change in dynamics in weeks eight to ten, where babies are more aware of other babies in the space so they sort of interact with each other. The mums are also much more comfortable in sharing in a sense, so I feel it almost felt like oh by week eight we finally really saw a difference, but then it was towards the end.” (S11) |
|  | Flexible and adaptable structure | “I think something particularly that I find useful as well is that there was like, you know, [X] started every week, sort of said, we’ll be here sort of half an hour also or come as early as you want, get settled, feed, let them finish feeding, the babies finish sleeping and things like that. And actually that really, really helped me because sometimes I would come 30 minutes early and just sit and chill and feed [X], have a drink or play with her a bit, or sometimes I'd have to just get the last minute because of how the morning had been or that sort of flexibility really helped my enjoyment.” (M1)  “I had a couple of sessions where I wasn’t able to talk for the whole session because of where I was at that day, and it doesn’t matter. You know, you just engage in the way that you want to engage with it, and I think that’s really nice.” (M18) | “I’ve been in one session once before where the artist just thought you’re all exhausted, just lie down, close your eyes, hold your baby or have your baby by your side and I’m going to sing to you, so it just gave them a chance to just not do anything and they didn’t have to do anything except be. So they completely changed the delivery of the session that week in response to how the mood was.” (S8)  “It is very intuitive work, you are kind of like completely just responding and you are responding to the baby, responding to the mums, responding to the room. Yes, and also I like to do things like hang around on a song… if that is feeling really good, then keep that going for a bit longer and then say if anyone has a harmony let’s add in a harmony… so treating it in a quite organic way depending on how the group seems to be responding.” (S9)  “I have, when I start, I’ve got a structure, you know, mum and baby songs, gentle songs, for mum songs, slightly more upbeat songs. But actually, as your week goes on, you can change that order. But, you know, baby might start crying so you can bring it back down again just to reset everything… It’s one of the freest ways I’ve ever worked in anything that I’ve done… you want mums to come in with no expectation, no stress, no anything and sometimes the mums have sung on their own but that has totally been organic. (S10) |
|  | Difficulty level of musical activities | “I think it was easy. So, I'm in the choir, so I’ve done a lot of singing before, so it was relatively easy in that sense. And then they’d break it down and we’d do repetition as well, so it was pretty much easy I think. But even for a novice it wouldn’t be too hard to catch on.” (M2)  “It was a bit more of a challenge I think doing that as well, it was really nice. And that challenge was something that I really enjoyed about it as well. You weren't just turning up and singing a song. You were turning up and learning how to sing different harmonies and things like that, and that was, yeah, I really enjoyed that.” (M3)  “It’s something that’s easy enough to do when you’re sleep-deprived and also focussing on a child, but also it’s kind of difficult enough to do that it kind of takes you out of yourself a little bit like the balance is just right.” (M5)  “I think I kind of struggle learning things just all like... Like just by listening, so I need to like read lyrics. So, when they weren’t in English, I found that quite difficult to remember.” (M7) | “Some of our leads try and encourage people to play around with lyrics which does actually work really well later on in the programme I think and some people will really respond to that but then others in the group find it too tricky with the words to remember and things like that because they’re still struggling a bit with their kind of memory… We have the lyrics available on the hub and I think if someone was really struggling each week with the lyrics we’d probably print them out and bring them to them.” (S6)  “I think from my perspective I do think it’s relatively… accessible. I think it’s the kind of skill of the artist again in that they don’t wade in on week one and try and get harmonies and rounds going, it’s all quite gradually built up and then without the mums quite knowing that that’s what’s happening.” (S8)  “If it’s too difficult I just move onto the next thing. But, to be honest, everything I teach is so simple anybody could learn it so it’s simple but satisfying because there are interesting harmonies and things.” (S13) |
|  | Diverse range of songs | “The songs were really good and they were … it was nice to have some that relate to sleep, some that are more I would say upbeat, and then some that are kind of just funny.” (M6)  “I really liked all the songs. There were some that were more calming and with loads of new like lullabies that I now sing... And then I really like the up-tempo ones as well... the song choice in terms of lyrics was really lovely as well, like all really positive and uplifting and there were a couple when I think the first time we sang them, I could feel myself welling up because they were just so sort of beautiful.” (M11)  “Some songs had words, some songs were in different languages, some songs came from sort of an African background so there was a lot more variety so I liked it from that perspective because it was something that I hadn’t done before. I liked it also that it felt also that it would be quite representative of different backgrounds as well so, you know, if you came from a Spanish background or there were some songs there that were sung in other languages so that was all positive.” (M14)    “I liked the way that there were international songs so just kind of singing in another language could be quite good because it’s just a different thing. I noticed that a lot of the songs had the theme of sunshine or spring or the sun and I thought that was a nice touch, it was good. But then there were the other songs that were more familiar like the Beatles of there was the Baywatch thing and it was a bit of fun and, yeah, I thought the content was really good.” (M20) | “So there was a Spanish mum, and for her she’d gone to some other sort of singing groups and had felt really discouraged but she didn't know the words and felt like that she, you know, felt like she was being in trouble for not knowing the words to these nursery rhymes and I think for her for everyone to be on a level playing field by it not being necessarily standard sort of nursery rhymes, the accessibility for non-English speakers, I think, or non, yeah, people that have grown up here, I think’s a real strength of the programme and obviously living in [X], in [X], that so many of the women we see won’t necessarily have grown up here. So, yeah, I think that was really nice strength of the programme.” (S7)  “So one is the kind of the diversity of songs that’s explored during a ten week programme, so really ensuring that that’s as diverse as possible so it’s not reliant on English as a first language, so to really make it accessible to women from different languages, different cultures. So making sure it’s quite rich and diverse but also explaining the origins of those songs so it really makes sense to women and it’s really transparent.” (S8)    “I think because we have a range of songs in different languages, different styles, you hope that you tick the box… It’s making it clear that the songs are kind of like overlapping for mum and baby, so, for example, a song that will challenge them that they could use as a lullaby.” (S10)    “I do international songs as well so I don’t just do English songs, because in London it’s quite diverse and it always like to keep that in mind.” (S13) |
|  | Singing together as a group | “I found it just so much fun I think because I guess it’s just that working together with other people to make something that you couldn’t make by yourself, right? So, when we were all singing in rounds and stuff it sounded wonderful.” (M5)  “I found it really uplifting, just singing – we were all saying… when they did the last session it is like that thing of singing together is really cathartic.” (M6)  “The first session I found really like amazing, like uplifting, sort of thing, especially when we’re all singing harmony and stuff like that.” (M7)  “I thought it was really nice, I had a really great sense of like progression, you know, as a group we sounded better at the end and that was satisfying so I thought it was spot on.” (M15)  “I really like doing the rounds because... it forces you to make eye contact with the people who are doing the round that you’re doing, so I know that when I’m withdrawing myself and I’m having a bad day, I really struggle to make eye contact. So, doing the rounds forces you to look up and to engage with people, and there’s something really terrifying about that but really lovely.” (M18) | “What I’ve found over the years is about halfway through, you do tend to get sort of what you would consider to be a breakthrough in terms of people’s confidence in singing, they suddenly realise how good they sound together as a group, so their confidence grows.” (S1)  “I think, yeah, the thing is that singing is going to produce endorphins, the sense of learning something together, the community, all of this stuff that we know singing is good for; so, that is what happens in the sessions.” (S13) |
| **People** | Small group size | I think the size of the group was good... I think there were probably seven mums and babies and for me that was good because I find larger groups less enjoyable... I think it’s easier to engage with a smaller group. It’s easier to get to know people. (M20) | “The size is so important for Melodies, the size of the group. What Melodies is so special about is obviously because it’s specific for those with postnatal depression, so that group is there, I think they did say it, you know, you’ll go to another singing group for mum and baby and the groups are massive. So, you lose that personal side.” (S10) |
|  | Shared mental health experiences among participants | “I think one of the things it made me realise that I'm not alone. A lot of mothers do go through what I have gone through and stuff, so and it's important that you can get a community around you and then you find people that are very similar to you.” (M2)  “I really liked having an activity where I knew that other mums would be sympathetic, I knew that they would also be struggling in some sort of way so it was fine to go along if I wasn’t feeling great that day and just be completely open.” (M8)  “I loved that it was in a community with other mums... who I know are in the same scenario, you know, that you don’t have to sort of walk into the room and be like, “Oh, I’m going to just like fake that I’m feeling really cheerful today”. People who are like... oh, I know these people get it so I can just be myself was really nice.” (M11) | “I think what is really good – and you can see quite quickly – is the fact that it is kind of a community, so bringing together people that may struggle with the same problems and I think it is still very taboo to talk about post-natal depression, that to have such a group and it was very liked because everyone came. Maybe they talk about oh my kid hasn’t slept all night or whatever, but it was in a way that other mums could relate.” (S11)  “I think that there’s been a couple of times when there’s been, you know, a much more vulnerable person there and it’s always felt like they’re the odd one out, to be honest… like we had one group where there was a much more vulnerable mum there and she got upset about something because someone had accidently taken her seat in the circle and she left and she didn’t come back, and I found that really sad, really difficult because I felt like I’d... it was my responsibility to make sure that didn’t happen.” (S2) |
|  | Shared activity between mother and baby | “I found it enjoyable because I think it was about me as much as it was for [X], my baby. I felt we were both equally included in the activities rather than me being her enabler to, other sort of… baby activities.” (M1)  “I just loved the singing, like she continued to respond really, really well to the singing like especially the lullabies, which was really great to have good lullabies.” (M15)    “He’d engage like banging the drum and shaking the shaker and it was really nice to feel that you’re slightly introducing them to music; that was really good, yeah.” (M20) | N/A (participant theme only) |
|  | Structured social time during sessions | “It was nice the way they… I think right at the beginning we didn’t… I think at some point they did a few things to kind of break the ice for everyone and that was wonderful, and we did bond quite a bit as a group I think. And so it was nice to have those kind of going round at the beginning just checking in. Some weeks it was like I'm not going to say I'm really tired again.” (M4)  “I think maybe if there was like more time either at the beginning or the end for like social interaction or something maybe would be good… yeah, just like more guided social time… Either like a break in the middle or like at the beginning or end.” (M7)  “I think we all felt that less time actually singing but more time just informal socialising would have been really nice, because actually an hour is quite a long time actually to just sing... it was really nice to get to know each other a bit more, have a chance to talk about whatever problems we were having at the time. So, in some ways it might have been nice to have 15 minutes to chat or something at the start then half an hour of singing and then 15 minutes at the end.” (M8) | “The kind of way that works, occasionally in the group itself, you might get a very, you know, a fairly innocuous question like, “How has your baby been this week?” which is what the musician will often do before the session starts. That sometimes spills over into something, like, they might say, “Oh, my baby hasn’t slept the whole week and I haven’t consequently slept the whole week, but I’m really tired, and I’m…” …and somebody else will say, “Oh, I’ve had a terrible week,” and then it becomes a little kind of group sharing, which is kind of tricky to contain... So we try and focus them, bring that back into, “Let’s do some singing, let’s kind of do something that’s very therapeutic in a vocal way.” (S1)  “I am not super-purist about it just being the music and there being no conversation – I add in little ice-breakers kind of thing, like I don’t know it can be as mundane as how are you and baby today, briefly tell us how you and baby are today and tell us who your favourite pop group was when you were a teenager, or something like that. Just little initiations to get them talking and using their voice in the group, I think really helps with them feeling settled in the group and bonding.” (S9) |
|  | Unstructured social time outside of sessions | “I mean we are not super active on our group. I mean we're not that local to each other, but I have bumped into a mum since and that was lovely and very friendly, and I've also been in touch with another one because we were sort of like talking about book recommendations. So I guess that's been another benefit.” (M4)  “I guess it was difficult, I don’t know if it’s been more successful with other groups, but our group didn’t like talk to each other that much, I guess… I guess everyone’s going through their own stuff but it just felt like we turned up and then went home. And there was like one mum that I once talked to on the bus ride home and I was like, oh, that was nice, maybe I’ll see her again, and then she just seemed to like not get on the same bus as me going back so we didn’t leave at the same time.” (M7)  “We had some WhatsApp group and now we are in touch... we are going to see each other next week. We are going to the park with our babies, so we are sharing some information about things we can do with our babies.” (M10) | “I don't think there's anything really that you can do about it, but then they did start a WhatsApp group afterwards but that’s just not really come to anything. And she’s tried to meet with them since. And it hasn’t really worked out. And I guess that was a bit of a shame as in it hasn’t for her, it hasn’t led to sustainable friendships, which is perhaps what she, I think she was just really incredibly lonely. But I'm not sure there's really anything that this mums’ group can do about that... It would be lovely to have some kind of ongoing support or something that we could offer because there definitely seemed to be the desire for it anyway from the mums that I've spoken to.” (S7)  “Yes, so that day for instance I saw lots of them leaving together, so little groups of three whereas they generally didn’t do that, so they were definitely becoming buddies and working out where they could walk together or whatever. Definitely people who maybe weren’t making as much eye contact at the start or feeling … as relaxed and present became very present in the group and in the room.” (S9)  “And I think one of the things that is really great is that they have a WhatsApp group attached to it so they can kind of chat. And I noticed that as soon as they brought that in... it meant that people were chatting and asking each other questions about stuff and then when they came the following week they bonded much more.” (S13) |
|  | Skills and values of music lead | “I did love it, and they made you feel like you could, you were actually a really good singer, where I’m sure I’m completely tone deaf, but they didn’t make you feel like that, it was very inclusive and lovely. And I described it, actually, yesterday with friends, as like a warm hug, like a metaphorical warm hug.” (M17)  “So, I thought that [X] the leader, the group’s singer, I just thought she did have the right kind of level of engagement. She was really supportive. You did feel that there was that extra support there from her to make us feel good about ourselves, which was really nice.” (M20) | “And it’s partly feasible because of the artist we have, so making sure that they are equipped and skilled and confident to be delivering in that way and with that kind of vulnerability in a group. (S8)  “We had a lady who said that she had been told at school that she had a bad voice and she shouldn’t sing as a child, and that was something that obviously I kind of dispelled, and said that is ridiculous… I would then really encourage the ones who felt less confident as singers, and say I don’t know little things like oh I am really glad I am sitting next to you, you sang that beautifully, it was really lovely to hear your voice today... So yes, warmth and kind of empathy and vulnerability myself, so if I mess something up, kind of laughing it off with the group. Often I would make a mistake on a round and bring them in at the wrong time and then we would all laugh about it.” (S9) |
|  | Support from additional staff | “The staff are so brilliant and so good at putting us at ease and making us feel welcome and relaxed… You get to a session and say, “I need to go for a wee, can you keep an eye on [X]?” And the staff will engage with [X] and really happy to help us if we need a drink or hold the baby or help get stuff out of the buggy and things and that made such a massive difference to me as well, because it made me feel like… I could ask for help.” (M1)  “I really thought the staff were just amazing, the Breathe people were so nice and so welcoming and just going to see them every week was so good. And also there just were like little snacks for us and [X] would go around giving us snacks and drinks. So, it felt like just this wonderful hour that was just for me to relax and have everything that I needed.” (M5)  “When me or other women would come in and our baby would be a little bit unsettled or a bit difficult, or, you know, we’d just be looking like we’d had a bit of a stressful day, they would literally just come over really serenely and just kind of say, “Hey [x],” and just take [x], my daughter, off me, and just, like, “Oh, I’ll just walk around the room with her,” and then she stopped crying and it gave me a minute to breathe. It was just those small gestures went a huge, huge way.” (M17) | “The need for that role of like the [X] in the room is so important because, you know, when you’ve got screaming twins that won’t stop crying like what’s the music lead supposed to do about that on their own whilst holding the session? You have to have that extra person in the room because especially with the group of women we’re working with like that’s a very stressful situation and so you need someone to be able to respond to that.” (S6)  “I wouldn’t deliver something that was signposted for maternal mental health without a team around me, because I am not experienced enough and I think for one person to carry that would be too much responsibility… Yes, I think having the Breathe member of staff there is critical and it also … [X] offers so much pastoral care to the mums as well, outside of the singing, which is an essential part of it. And, I couldn’t deliver the way I am delivering if I was … even just like basic things, like having to check phone messages of people who are running late or … yes, so I think it is just so invaluable. They also are such warm, compassionate people so it is really important.” (S9)  “I think the thing is first and foremost the fact [X] is incredible, like looks after. They never, ever feel shy to say, “Can you just watch the baby while I go for a wee?” you know, things like that which they feel comfortable there.” (S10) |
| **Contexts** | Location and transport | “Like it is a bit of a walk for me, and actually I think that was really good. It made me go out and I had a good 35-minute walk there, 35-minute walk back… I guess the only other thing was the location of the sessions meant that often me and the other mums would walk and talk to each other on our walks home. I was just thinking I wonder if the sessions were in a location where there was somewhere nearby, whether we maybe would have stuck around and said, “Oh does anyone want to go for a coffee?” or if there was a park nearby.” (M1)  “It was quite far away for me so maybe it was a 35 – 40 minute walk, but that was fine because she’d normally have a sleep on the way and I quite enjoyed the walk actually… If it was up to me I would have this in every children’s centre and it would be nice, because I’d imagine maybe having that distance to travel might put some people off if it’s a bit more difficult. And I know some people did come from further away.” (M5)  “I mean I could actually have walked in an hour which I would have done if it was a nice walk. But, it was just so horrible, the walk is just quite a depressing walk and really busy roads. And, the same I could take two buses, but I just found the first – I did that the first couple of times and they were full, and I just found the journey quite depressing!” (M8)  “It only took 40 minutes I’d say which, you know, for London everything’s about 40 minutes from everywhere but it was more that it was the… tube and then I had to change so it was sort of, you know, it sort of was more faffy than it was… it’s much more stressful than you think and especially the tubes in London they’re not so straightforward.” (M15) | “I did a referral to Melodies for Mums that was outside of our locality, and I think because specifically with this mum she had a bit of a financial struggle so what she found was that was a challenge for her to commute on public transport to go to a different centre. But, she overcame that so she was able to do it but that the only kind of challenge that she had in the past, yes.” (S3)  “I think from memory what was quite good is that you did have quite a lot of different options it different children’s centres or different places, so it felt like there was always one local to where the mums live sort of thing.” (S7)    “Well, I think what was good for most mums is that it was a walking distance so just because I know there was a tube strike at some point, so I couldn’t come, but it was mostly mums in the area so they could still come, so that was good.” (S11)  “There’s anxieties, there’s lots of fear, there’s fear of how to get around with your baby in a pram. So, transport can be a real challenge, so trying to find a venue that has lots of different options to get to it, so there might be good bus links, train links, tube links and walking routes that feel safe, that can be quite challenging.” (S12) |
|  | Time and day of sessions | “Only when I started changing my son’s nap routine. It was a little bit of a challenge to fit it in around that, but I mean, you know, all babies have different nap times.” (M3)  "I guess if he hadn’t been at nursery on the day that the programme was running… I wouldn’t have been able to do it… Yes, I couldn’t have – he is pretty demanding so it is not as if I could have brought him along and put him in the corner to read there! Yes, and it would have been far too expensive to pay for childcare.” (M8)  “It wasn’t a problem for us, but the time of day could be a bit tricky in terms of nap times because we had 1.30 till 2.30, which, if you’re trying to get into a nap schedule, would be when you’d want... probably when you put baby down for a nap.” (M11)  “I have another child so I had to leave at 2:30 to go and collect her from school. And her school finishes at 3:30. So, if any mums had kids that finished at 3 they might have found it really difficult.” (M20) | “Another challenge is it hasn’t come up loads but it has come up once or twice is if a mum has other children so if this is their second or third child childcare being a preventative from being able to take part, so if they’re not in nursery or school yet, you know, Melodies is for the baby up to nine months and the mum whereas having another sibling in the room would really, really change that dynamic of a group. So we have looked in the past at can we arrange childcare for that additional sibling? Would they be in the room next door? Then we need another space. It gets quite logistically challenging but it hasn’t come up that much but it has come up.” (S8)  “I think the one thing is in terms of feasibility, having to find this one-hour slot for all mums is tricky because it clashes with feeding or naps and everything, and every mum has a different structure of the day. So, I know one mum would kind of come in 30 minutes later, because her babies were napping and obviously you kind of want the babies to also take part as much as they can, and not just stay in the corner and sleep.” (S11) |
|  | Recruitment and advertising | “I have no idea how you would go about communicating it and putting it out there to people, because again when I think about it I would have not have known it existed – it was only because someone had casually mentioned it and I looked it up.” (M9)  “As far as advertising it my friend sent it to me. I don’t think I would’ve found about it otherwise apart from my friend sending it to me. I don’t know how it’s advertised or anything and I don’t use social media so obviously if it’s on social media then I wouldn’t see it… Can health visitors hand out leaflets and heavily recommend? Because I didn’t get any of that information from a health visitor, so if that kind of came in a pack and it’s free, then I wouldn’t see why they wouldn’t push it.” (M17)  “I was really just interested to see how this would work in terms of making it more accessible to people who might not necessarily know how to get involved with these kinds of things, especially if you’re in a particularly bad place, you’re not really up for sitting online and doing lots of searching and you’re probably also withdrawn from people and not necessarily talking, because a lot of the time you find out about these things by word of mouth.” (M18)  “It is hard, isn’t it, how to reach people particularly people that are experiencing low mood, they might just be at home a bit more reluctant to engage anywhere, they might not have been to a children’s centre. And different demographics and stuff because it was quite a white middle class thing. I mean it wasn’t exclusively but most people were and you think it could be opened up to a lot more different people probably, but it’s just how to reach them yeah.” (M20) | “I think recruitment is a big challenge and I think the other issue with that just more broadly is that because postnatal depression, you know, is less of a stigma in this country than others but there’s still stigma around it, there’s still potentially like people don’t know they’re experiencing it actually and that kind of recognition of what they’re experiencing, it makes it harder to reach those people I think… So, again we’ve adapted it so that we’ve like used different language that maybe might be more relatable like things like anxiety and low mood are stuff that’s more relatable than you’ve got PND like it’s just that’s how we’ve got around that. And again, I think it’s all these things that we’ve sort of adapted to make it more effective but they are challenges nonetheless.” (S6)  “We’ve tried quite a lot with link workers or the link workers, social prescribers but I just don’t think that works because women don’t end up referred to them, so if women have symptoms of postnatal depression they tend to be under a GP care or health visitor so that kind of strategy of really promoting it to link workers and social prescribers just hasn’t really paid off. They tend to be more general mental health referrals or isolation rather than something quite specific like postnatal depression, so that hasn’t worked and I don’t think it’s worth us pursuing much in the future, it’s just a learning I guess for us to where referrals go within the healthcare system and they don’t end up there most of the time.” (S8)  “Maybe recruiting from what I heard was a bit difficult, just because there is not that much awareness about mum’s mental health I feel, and also legitimacy of creative therapies… I mean I guess I don’t know how they sort of phrase it because I know sometimes it is mums with post-natal depression and it is quite a big diagnosis, that is still not very much acknowledged.” (S11) |
